# The genome trilogy of *Anopheles stephensi*, an urban malaria vector, reveals structure of a locus associated with adaptation to environmental heterogeneity

**DOI:** 10.1038/s41598-022-07462-3

**Published:** 2022-03-04

**Authors:** Aditi Thakare, Chaitali Ghosh, Tejashwini Alalamath, Naveen Kumar, Himani Narang, Saurabh Whadgar, Kiran Paul, Shweta Shrotri, Sampath Kumar, M. Soumya, Raksha Rao, Mahul Chakraborty, Bibha Choudhary, Susanta K. Ghosh, Suresh Subramani, Sunita Swain, Subhashini Srinivasan

**Affiliations:** 1 Institute of Bioinformatics and Applied Biotechnology, Biotech Park, Electronic City Phase I, Bangalore, 560100 India; 2 Tata Institute for Genetics and Society, Center at inStem – GKVK campus, Bellary Road, Bangalore, 560065 India; 3 University of California, Irvine, CA 92697 USA; 4 University of California San Diego, La Jolla, CA 92093 USA; 5 National Institute of Malaria Research, Bangalore, 562110 India

**Keywords:** Computational biology and bioinformatics, Evolution, Genetics

## Abstract

*Anopheles stephensi* is the most menacing malaria vector to watch for in newly urbanising parts of the world. Its fitness is reported to be a direct consequence of the vector adapting to laying eggs in over-head water tanks with street-side water puddles polluted by oil and sewage. Large frequent inversions in the genome of malaria vectors are implicated in adaptation. We report the genome assembly of a strain of *An. stephensi* of the type-form, collected from a construction site from Chennai (IndCh) in 2016. The genome reported here with a L50 of 4, completes the trilogy of high-resolution genomes of strains with respect to a 16.5 Mbp 2R*b* genotype in *An. stephensi* known to be associated with adaptation to environmental heterogeneity. Unlike the reported genomes of two other strains, STE2 (2R+^b^/2R*b*) and UCI (2R*b*/2R*b*), IndCh is found to be homozygous for the standard form (2R+^b^/2R+^b^). Comparative genome analysis revealed base-level details of the breakpoints and allowed extraction of 22,650 segregating SNPs for typing this inversion in populations. Whole genome sequencing of 82 individual mosquitoes from diverse geographical locations reveal that one third of both wild and laboratory populations maintain the heterozygous genotype of 2R*b*. The large number of SNPs can be tailored to 1740 exonic SNPs enabling genotyping directly from transcriptome sequencing. The genome trilogy approach accelerated the study of fine structure and typing of an important inversion in *An. stephensi*, putting the genome resources for this understudied species on par with the extensively studied malaria vector, *Anopheles gambiae*. We argue that the IndCh genome is relevant for field translation work compared to those reported earlier by showing that individuals from diverse geographical locations cluster with IndCh, pointing to significant convergence resulting from travel and commerce between cities, perhaps, contributing to the survival of the fittest strain.

## Introduction

*Anopheles* (*An.*) *stephensi* is a malaria vector prevalent in urban India, South Asia, Middle East and, more recently, expanding its range into Africa as urbanisation continues^1^. The persistence of *An. stephensi* in urban settings is linked to its ability to lay eggs in overhead water tanks, when the stagnant water on the streets is rendered unsuitable^2^. The genomes of a few strains of *An. stephensi* have been reported recently, including that of a laboratory strain of *An. stephensi*, STE2, MRA-128, originally collected from Delhi by the National Institute for Malaria Research (NIMR) and subsequently maintained at the Walter Reed Hospital^3^. The STE2 strain has been extensively studied, leading to several publications including the complete physical map^4^ and chromosomal studies of several inversions via photomaps of loop formation, including the heterozygous 2R*b* inversion^5^. As early as 2014, using a cocktail of the then state-of-the-art sequencing technologies, this group sequenced DNA extracted from a pool of 50 lab-reared individuals to assemble a draft genome^6^. Several recent attempts improved this draft genome using synteny^7^, homology-based approaches^3^ and paired-end HiC reads^8^ to obtain near-chromosome level assemblies, which confirms the genotype of the 2R*b* inversion in this strain as the heterozygous form (2R+^b^/2R*b*)^3^. This report catalogued the majority of the coding genes and their structures^6^ in *An. stephensi*, for the first time.

More recently, a very high-resolution genome was reported for a laboratory strain, hereafter referred to as the UCI strain, providing a gold-standard reference genome in guiding future malaria research^9^. The contig-level statistics of this assembly is close to those of the human and fly genomes, the two organisms that are the most complete to date. Based on this assembly and reconciliation with HiC data, it has been reported that the UCI genome is homozygous for the 2R*b* inverted form (2R*b*/2R*b*)^3^.

As early as 1972, polytene chromosomes from ovarian nurse cells in adult *An. stephensi* mosquitoes were used to study the association of inversion polymorphisms with certain experimental or ecological situations^13^. At that time, the 2R*b* inversion was known as the Karachi variant (KR). Using *An. stephensi* strains from four distant geographical locations including Iraq, Iran, Pakistan and India, this group reported that the strains from India were homozygous for the standard form of the 2R*b* configuration (2R+^b^/2R+^b^); those from Iraq/Iran were heterozygous and that from Karachi was homozygous for the 2R*b* inversion (2R*b*/2R*b*). The inversion karyotype was correlated with early emergence for homozygous 2R+^b^/2R+^b^, intermediate emergence for heterozygous form 2R+^b^/2R*b* and late emergence for homozygous 2R*b*/2R*b* form. Also, it was reported that a cross between opposite homozygous forms did not produce intermediate emergence, suggesting a selection process that is more complex. They studied the feeding behaviour of 2R+^b^/2R+^b^, 2R+^b^/2R*b* and 2R*b*/2R*b* genotypes of Iraq strains and noticed differences in sugar-feeding propensity during photophase, implicating this region in the control of circadian rhythm.

The strain homozygous for the inversion (2R*b*/2R*b*) was also independently observed and reported in 1984 in individuals from Karachi^14^. They reported many offspring of a single female that were homozygous for the 2R*b* inversion, which is implicated in resistance to alphamethrin, which was coincidently approved by WHO for adult malaria control around 1981–1985^15^. They observed and reported several inversions across the genome of *An. stephensi* by studying polytene chromosomes from ovarian nurse cells of various strains from different locations. In this report, photomaps of both standard and inverted configurations of the chromosomes for several inversions were documented. Using visible loop formation near the inversion region and assigning banding patterns, they were able to identify/validate a number of inversions including three from the 2R arm, two from the 2L arm, three from the 3R arm and several from the 3L arm along with approximate breakpoints based on cytobands. There was no inversion reported in the X chromosome. They also demonstrated that, except for a few inversions, a significant number of inversions, catalogued in the wild population, was lost during the first few generations of rearing in the lab. However, among those that were persistent for many generations of rearing in the laboratory are 2R*b*, 2L*c*, 3R*b* and 3L*b*.

More recently, Kamali et al.^4^ used 12 microsatellite markers in conjunction with a collection of previously-known breakpoints to correlate the inversion status with phenotypes. In this report, using microsatellite markers on polytene chromosomes, it was shown that the E7T microsatellite marker, which maps near the 14C band within the 2R*b* inversion region, is diverse in the three forms of *An. stephensi* including type, intermediate and mysorensis.

The importance of paracentric inversions in *Anopheles* has been well established in *An. gambiae* and is shown to be associated with the ability of some vectors to follow humans in diverse environments^10^. By studying polytene chromosomes from 1500 individuals of *An. gambiae* from both the forest and the savannas, it was reported that those from the forest are homozygous for standard forms of inversions, but those from the savannas display complex inversion patterns in chromosome 2 of *An. gambiae*. Of these, the 2R*b* and 2L*a* inversions are the best-studied in terms of their frequencies associated with traits^11,12^. It was proposed that some inversions may be as old as the expansion of agriculture and may have been maintained in the population for a long time allowing for genetic drift between the heterozygous arms. The two breakpoints of the 2R*b* inversion are reported to contain repetitive regions, making it prone to structural and sequence level instability^16^. In the case of the 2L*a* inversion, by revealing the structure at the breakpoint, it is proposed that this inversion has descended from a single event^17^. Furthermore, the 1000 *An. gambiae* genome project also enabled extraction of SNP signatures useful in the association of 2R*b* inversion genotypes with individual mosquitoes^18^. Cost-effective technologies, such as RFLP-PCR, are being explored to type 2R*b* signature SNPs in *An. gambiae* from a large number of individuals^19^.

Here, we report the genome assembly of a type-form of *An. stephensi* collected from a construction site in Annanagar, Chennai, India (IndCh), which was reared in the laboratory since 2016. Unlike the genomes of the two other strains reported for *An. stephensi*, the IndCh strain is found to be homozygous for the standard form of the 2R*b* chromosome configuration (2R+^b^/2R+^b^), allowing us to extract segregating signatures and the identification of candidate genes responsible for the expansion range of this important vector.

## Results

### Assembly of the genome of IndCh strain

Using DNA extracted from 60 individual iso-females homogenised by sib mating over five generations, we generated both 60X coverage of PacBio reads using Sequel 1 technology for an average length of 9000 bp and 100X coverage of Illumina reads. PacBio reads were assembled using multiple tools, including Canu^20^ and FALCON^21^ to get contig-level assemblies with L50 values of 20 and 28, respectively. The two assemblies were merged using Quickmerge^21^ to obtain an improved, contig-level assembly with a L50 of 8. The merged assembly was corrected for sequencing errors using two rounds each of Arrow and Pilon polishing^22^. The statistics of these assemblies are listed in Table 1.

**Table 1:** Statistics of the genome assembly of the IndCh *An. stephensi* strain.

Assembly stage	No. of contigs	Longest Contig (Mbp)	N50 (Mbp)	L50	Assembly size (Mbp)	Average Contig Length (Kbp)	Median Contig Length (Kbp)
Canu assembly	1683	10.7	3.0	20	219.8	130.6	10.9
FALCON assembly	1250	9.7	1.8	28	231.1	184.9	21.2
Merged and polished	1010	26.5	8.5	8	225.8	223.6	19.0
HiC scaffolds	962	35.9	21.4	4	226.9	236.0	18.6

**Figure 1:**
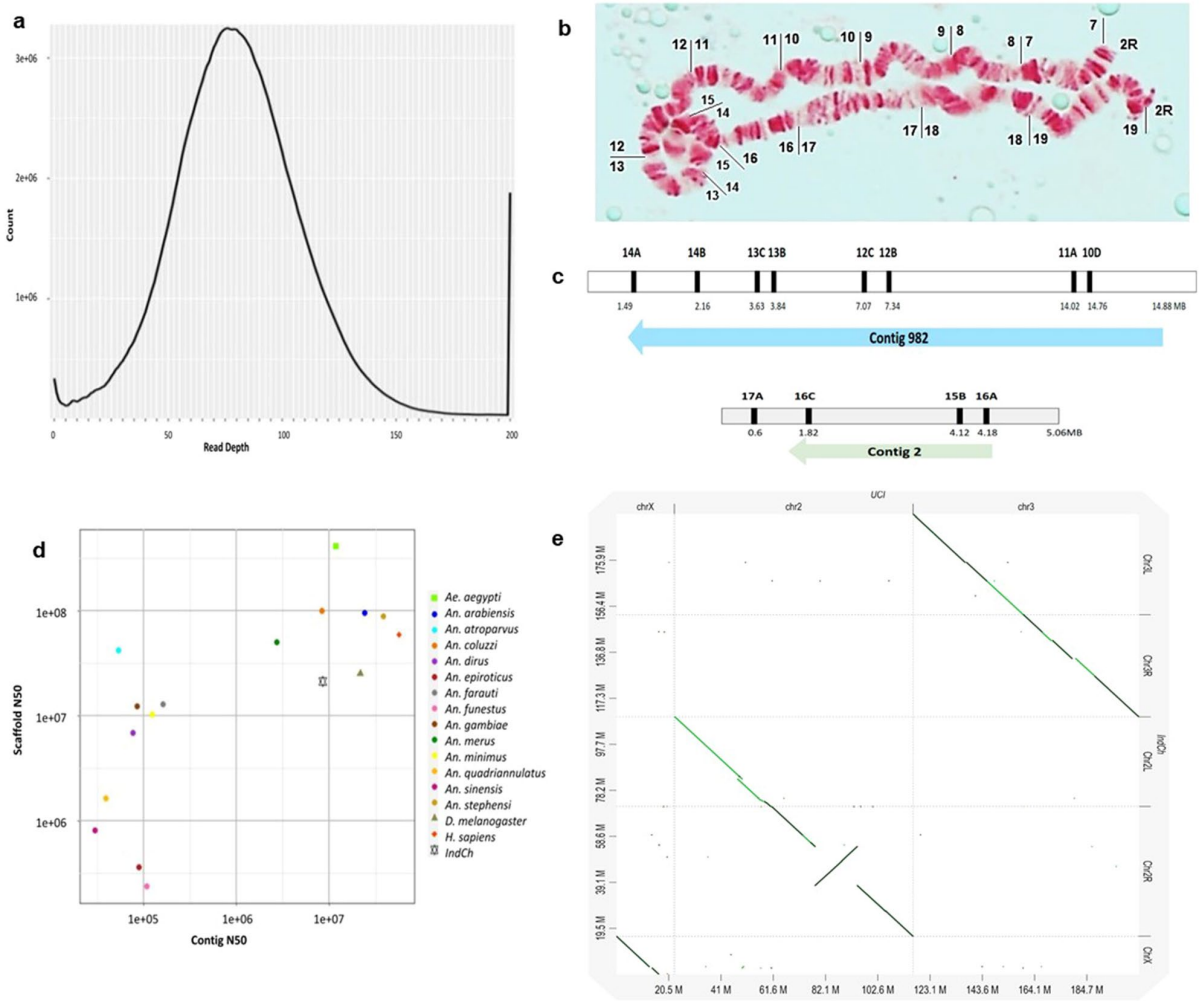
Assembly features of IndCh genome: (**a**) Coverage analysis for the assembly of the IndCh strain showing single, instead of the double hump as expected for heterozygosity in the 2R*b* inversion. (**b**) Photograph of polytene chromosomes from the IndCh strain showing the standard configuration of 2R*b* as found in 96% of individual mosquitoes in the outgrown population. (**c**) Two contigs in support of the standard form of 2R*b*, for example, Contig-982 (top) supports the distal breakpoint near physical marker 11A to 12B and contig-2 (bottom) supports the proximal breakpoint near marker 16C to 17A. (**d**) Comparison of the assembly statistics with other organisms including *Anopheles* species, fly and human genomes. (**e**) Dot-plot of IndCh assembly against the UCI genome.

In order to select the most relevant publicly available HiC dataset for building scaffolds for the IndCh strain, an attempt was made to establish the 2R*b* inversion status for the IndCh assembly. For this, sequences of physical markers corresponding to chromosome 2R arm from Jiang et al*.*^6^ were aligned against the merged, contig-level assembly mentioned above. The markers spanning the breakpoints for the 2R*b* inversion were distributed on 2 different contigs (contig-982 and contig-2). The linearity of the physical markers from 11A to 12B and 16C to 17A, consistent with 2R+^b^/2R+^b^ configuration were both found within the two contigs shown in Fig. 1c, indicating that IndCh supports the standard configuration of 2R*b*. There were no contigs supporting the inverted form of 2R*b* in which one expects 11A followed by 16C on one side and 12B followed by 17A on the other. Furthermore, observation of polytene chromosomes from ovarian nurse cells suggests lack of heterozygosity in the 2R arm (Fig. 1b) in 96% of the 61 insects bred over 35 generations after the iso-females were selected for sequencing (Fig. 7). Haplotype phasing of the assembly of the IndCh strain was done using a set of primary contigs and a set of associated contigs from FALCON and FALCON-unzip^23^ tools, respectively. The lack of a double peak in the IndCh phased contigs (Fig. 1a) along with other data mentioned above, supports that the IndCh strain has the standard homozygous form of 2R*b (2R*+^*b*^*/2R*+^*b*^*)*.

The HiC data from the STE2 strain, which is heterozygous for the 2R*b* inversion, contains reads representing the standard form of 2R*b* and were selected for scaffolding the merged contigs of IndCh^3^*.* A scaffold-level assembly with a L50 of 4 was obtained for IndCh (Table 1). These scaffolds were assigned both positions and orientations on the chromosomes using physical markers^6^ and were stitched into pseudo-chromosomes (Supplementary Fig. 1). Figure 1d compares the quality and completeness of the assembly reported here to those reported for other *Anopheles* species, the fly and the human, suggesting that the IndCh assembly is comparable to the other high-quality genomes at both contig and scaffold levels. A dot-plot between all the chromosomes of IndCh and UCI assemblies clearly shows the 2R*b* inversion in the UCI genome (Fig. 1e).

### Interrogation of 2R*b* breakpoints

**Figure 2:**
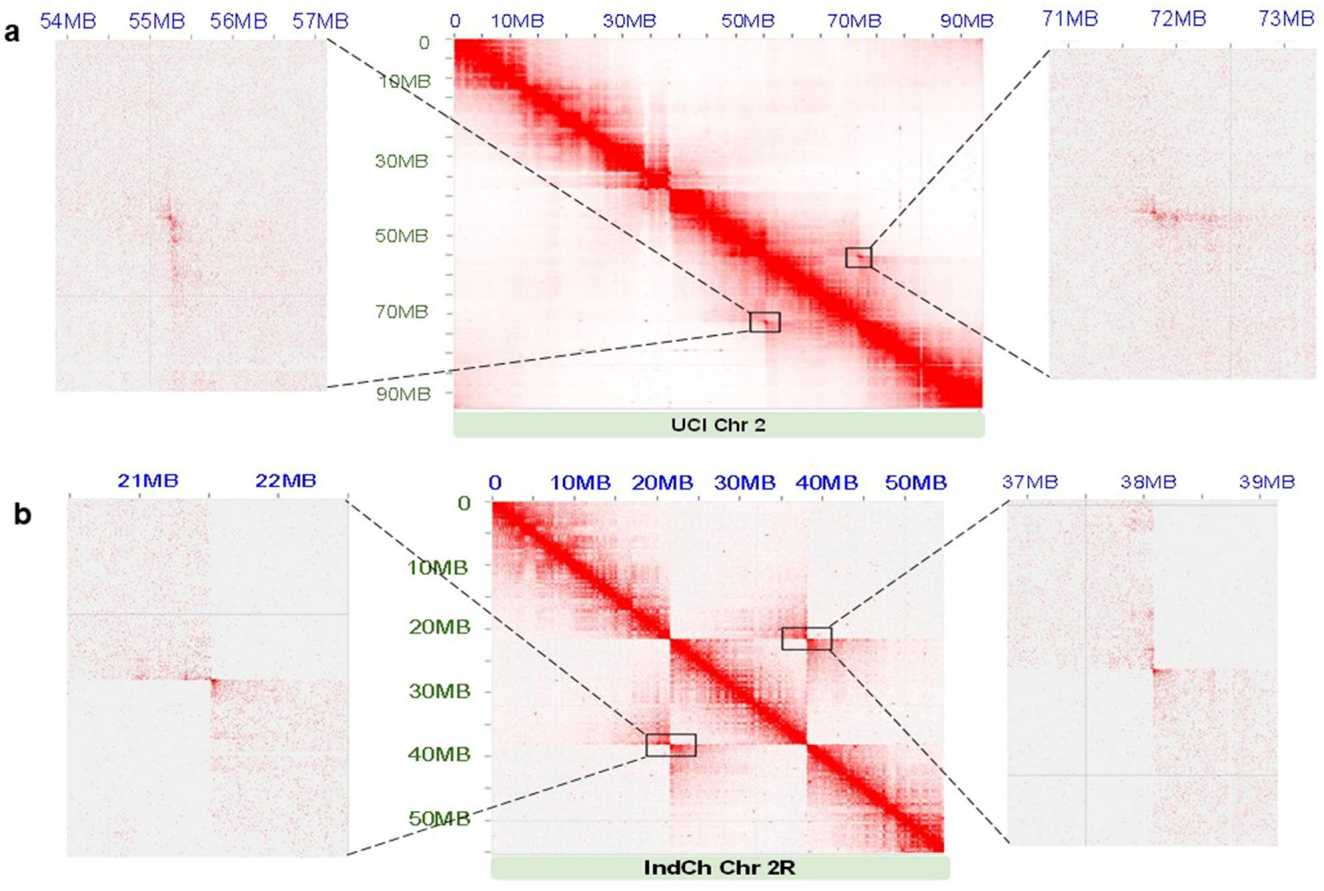
Interrogation of 2R*b* breakpoints: (**a**) Proximal and distal breakpoints obtained by mapping STE2 HiC against the chromosome 2 of UCI. The black box represents the proximal and distal breakpoints located between the ranges of 55,265,000–55,270,000 bp and 71,795,000–71,800,000 bp, respectively, in the UCI genome. Adjacent to the contact map are the enlargements of the butterfly structures, for a resolution of 5 Kbp per dot. (**b**) Proximal and distal breakpoints obtained by mapping the UCI HiC against the chromosome 2R of IndCh. The black box represents the distal and proximal breakpoints located between 21,510,000 to 21,515,000 bp, and 38,085,000 to 38,090,000 bp, respectively, in the IndCh assembly. Adjacent to the contact map are the respective enlargements of the butterfly structures for a resolution of 5 Kbp per dot in the IndCh assembly.

With high-resolution genome assemblies of the IndCh (2R+^b^/2R+^b^) and the UCI (2R*b*/2R*b*) strains and public availability of HiC data from two strains with the different 2R*b* configurations, an attempt was made to define the 2R*b* breakpoints for *An. stephensi* at the sequence-level. The rationale was to map the HiC reads from a given strain onto the assembly with the opposite 2R*b* configuration to produce off-diagonal butterfly structures. For example, HiC data from the STE2 strain, which is heterozygous for the 2R*b* inversion, but contains reads representing the standard 2R*b*, was mapped to the genome of the UCI strain (2R*b*/2R*b*) (Fig. 2a). In this case, the read-pairs making the butterfly support the standard form of 2R*b*. On the other hand, the HiC data from the UCI strain that contains reads supporting only the homozygous inverted form (2R*b*/2R*b*) was mapped onto the assembly of IndCh (2R+^b^/2R+^b^)*,* creating two very sharp butterfly structures corresponding to two breakpoints in the IndCh assembly (Fig. 2b). The coordinates of the centre of the butterfly in both contact maps were deciphered to a maximum resolution of 5 Kbp per dot by enlarging the contact map. Using this approach, the region of 2R*b* breakpoints in the UCI genome were estimated to be between chr2:55,265,000–55,270,000 on the proximal end, and chr2:71,795,000–71,800,000 on the distal end (Fig. 2a). Likewise, the loci of the 2R*b* breakpoints in the IndCh assembly were estimated to be between chr2R:21,510,000–21,515,000 on the distal end, and chr2R:38,085,000–38,090,000 on the proximal end (Fig. 2b).

In order to further refine the breakpoints**,** the error-corrected PacBio reads of IndCh were mapped onto the chromosome 2 of the UCI genome. Since the UCI and IndCh strains are of opposite genotypes with respect to 2R*b*, one would expect a gap among IndCh reads near the UCI breakpoints, (Supplementary Figs. 2a and 2b, top panel). Based on the location of the gap, the proximal breakpoint on the UCI genome was refined to chr2:55,256,667–55,257,873, which is 10 Kbp or two dots (5 Kbp each) left of the estimated breakpoint from the contact map. Similarly, based on the location of the gap, the distal breakpoint was refined to chr2:71,807,500–71,808,000, which is also 10 Kbp or two dots right of the estimated breakpoint locus**.** Furthermore, mapping of Illumina reads from IndCh onto the genome of UCI, confirmed the refined breakpoint region (Supplementary Figs. 2a and 2b, bottom panel). Additionally, in support of the IndCh assembly near the breakpoints, both error-corrected PacBio and Illumina reads from IndCh mapped onto the IndCh genome showed no gaps (Supplementary Figs. 2c and 2d), thus validating the assembly near the standard 2R*b* breakpoints.

### Characterising chromosome breakpoints

**Figure 3:**
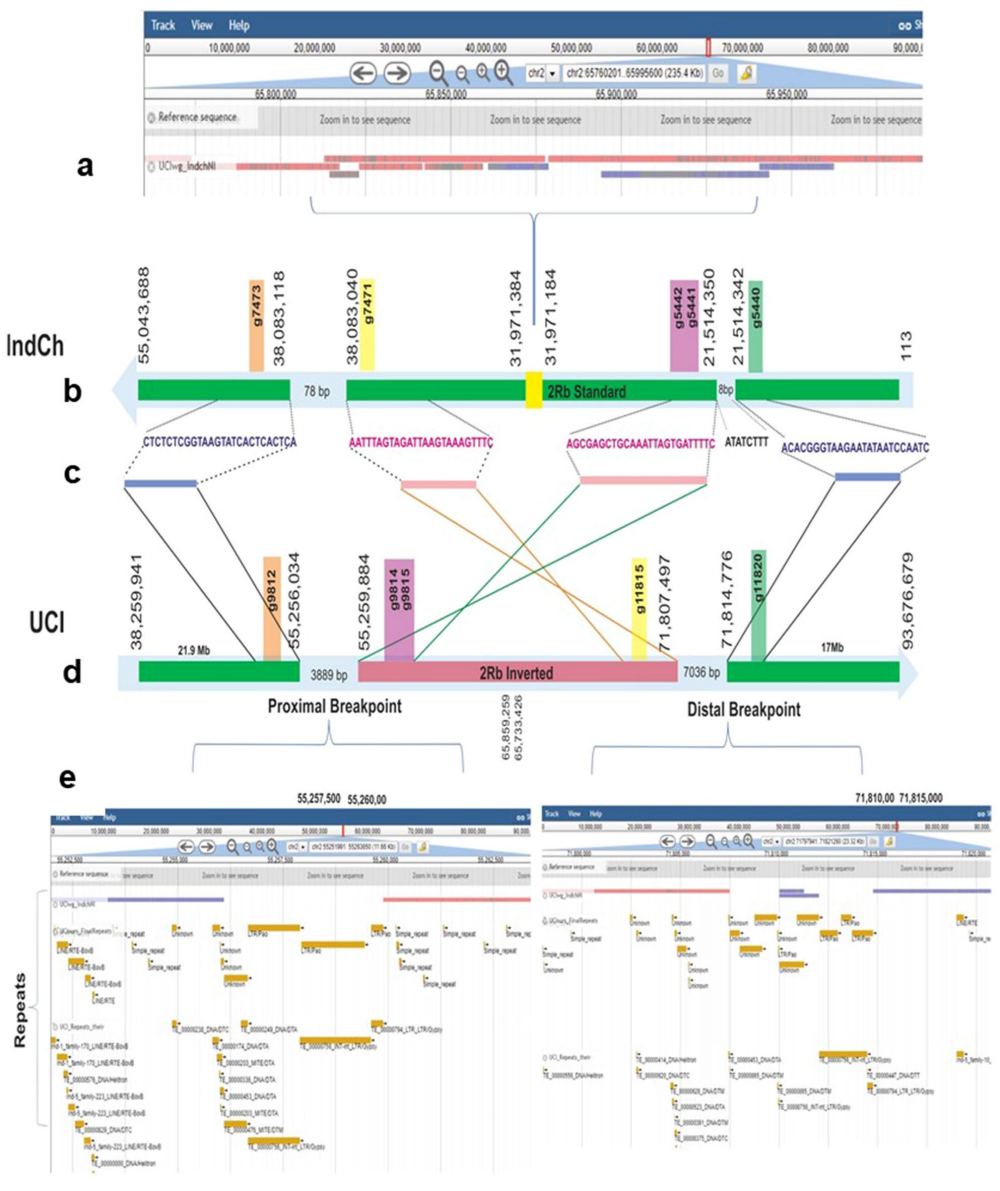
Characterisation of the IndCh and UCI assembly: (**a**) a schematic of unimap of the IndCh assembly on the UCI genome showing the overlapping segments in the middle of the 2R*b* inversion. (**b, d**) IndCh and UCI blocks, respectively, that are homologous with chromosome coordinates at the start and end of each block. (**c**) Highlights the sequences near the breakpoints shown by blue boxes (standard orientation) and the pink boxes (inverted orientation), (**e**) repeat elements in the gap between breakpoints in the UCI assembly.

The IndCh assembly was mapped to the genome of UCI using ‘unimap’ (https://github.com/lh3/unimap). There are three major blocks from the 2R arm of the UCI genome with extensive homology with the IndCh assembly, as shown in the schematic in Fig. 3b, d. On the two sides of the 2R*b* inversion, the region ~ 71.8 to 93.7 Mbp of the UCI 2R arm maps to the region from the beginning of the 2R arm of IndCh through 21.5 Mbp. Similarly, the region from ~ 38.3 to 55.3 Mbp of the UCI 2R arm maps to a region from ~ 38.1 to 55.0 Mbp of the IndCh assembly. Within the 2R*b* inversion, at coordinate ~ 66 Mbp in UCI and ~ 32 Mbp in IndCh, there is significant duplication and minor inversions in IndCh, as shown in Fig. 3a. The homologous regions inside of the 2R*b* inversion in both the assemblies are around 16.5 Mbp and the regions outside of the inversion are nearly 16.9 Mbp (proximal) and 21 Mbp (distal) in both the assemblies. On the other hand, near the breakpoints, thousands of extra bases are found in the genome of the UCI strain with very short stretches of corresponding sequences in the IndCh assembly, as shown between blocks in Fig. 3d. Interestingly, these extra bases are filled with repeat elements, as shown in Fig. 3e.

Figure 3c shows the homologous sequences near the breakpoint between the IndCh and the UCI assemblies as determined using the BLASTN tool. We also found orthologous genes (colour coded in Fig. 3b, d) on both sides of the two breakpoints that are consistent with the inversion genotype. In the IndCh assembly, the homologous sequences spanning the breakpoints are interrupted by short stretches of non-homologous sequences shown in the Supplementary Text 1 and 2 and have been validated using PCR and de novo assembly of an intermediate strain IndInt (PRJNA757559). As shown in the Supplementary Fig. 3a, one of the primer pairs amplified the proximal breakpoint from individuals from the IndCh population outgrown for 35 generations and another primer pair amplified the distal breakpoint (Supplementary Fig. 3b) from the parent population from which the IndCh iso-female line was originally generated (Fig. 7).

### Genes within the 2R*b* region

**Figure 4:**
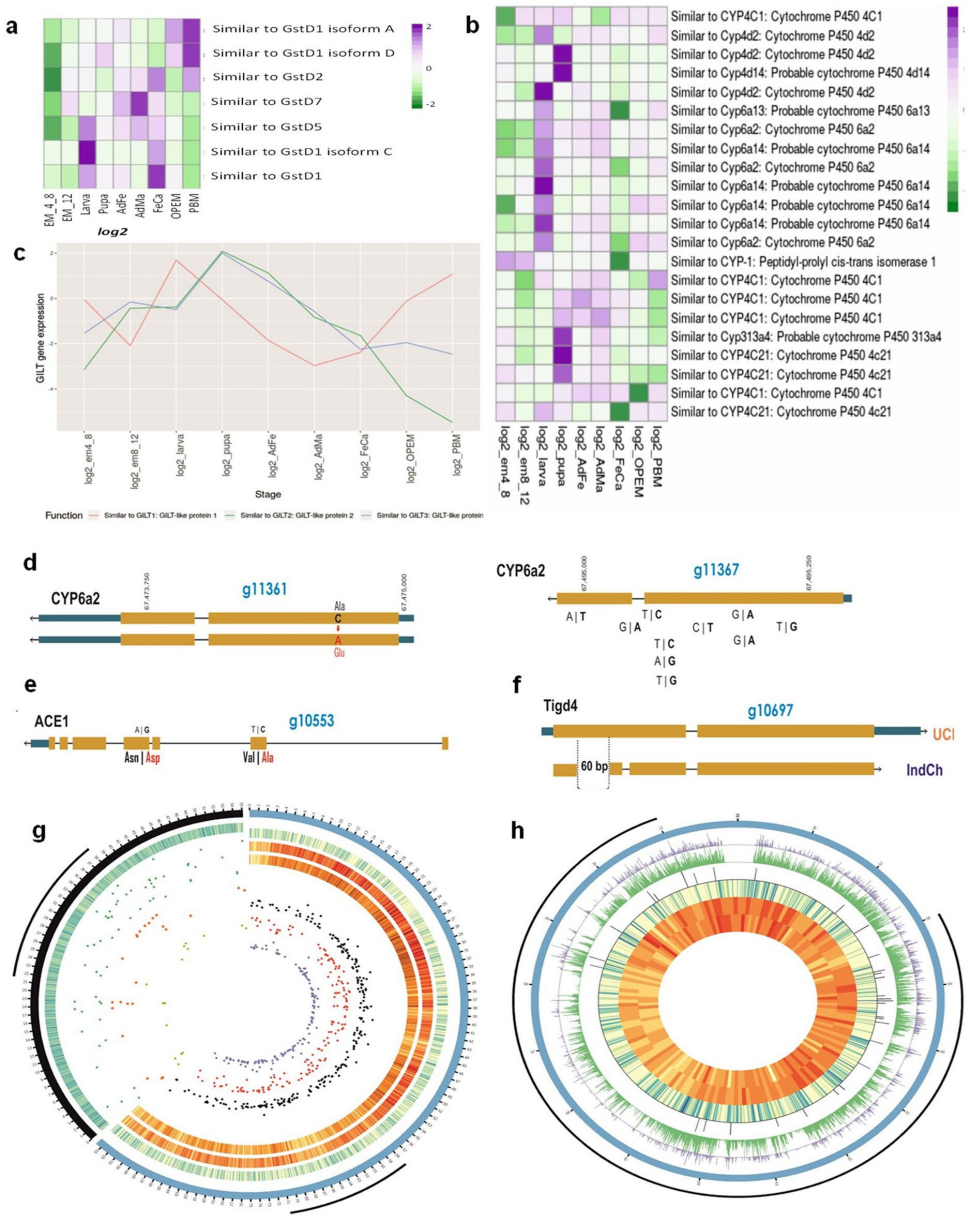
Genes within the 2R*b* region: (Key : EM_4_8—Embryonic stage from 4 to 8 h; EM_12—Embryonic stage at 12 h; OPEM—Ovaries Post Emergence; FeCa—Female Carcass; AdMa—Adult Male; AdFe—Adult Female; Larva—Larva stage; Pupa—Pupa stage). (**a**) Expression profiles of GST paralogues across developmental samples. (**b**) Expression profiles of Cyp450 paralogues across developmental samples. (**c**) Expression profiles of GILT paralogs across developmental samples. (**d**) Missense SNP in Cyp6a. (**e**) Two of the four missense SNPs in the ACE1 gene. (**f**) The gene Tigd4 with 60 bp deletion in the middle of the first exon. (**g**) (from outside to inside, left to right): The outermost black arc represents IndCh Chr2R and the blue arc represents UCI Chr 2; the heat map represents the gene density; the green dots are insertions; the orange dots are the LTR-Gypsy elements; the yellow dots are the genes intersecting with the inserted LTR-Gypsy elements in the IndCh strain. The red dots are the LTR-Gypsy elements present in the UCI strain; the black dots are the intersecting SVs that are deleted in the IndCh strain; the purple dots are the genes intersecting with the LTR-Gypsy elements and SVs in the UCI strain; the three adjacent heatmaps show the density of deletions, insertions and genes, respectively in the UCI strain. (**h**) (From outside to inside) The black arc represents the 2R*b* region in the UCI strain; the blue circle is chromosome 2 of the UCI strain from 55 to 72 Mbp; the purple peaks represent 3781 significant SNPs with a p-value of 0.0005 among the candidate SNPs for 2R*b*, the green peaks represent the 22,650 candidate SNPs for 2R*b*, while the black peaks are the densely segregating SNPs (277) from the selected blocks (7 or more SNPs in a 1500 bp region). Followed by these are the tracks for the density of genes, insertions and deletions, respectively.

The 2R*b* region in *An. stephensi* spans 16.5 Mbp and has 1353 predicted genes, including ACE1, as well as tandem clusters of GST and Cyp450 paralogues implicated in insecticide resistance. This locus also includes 3 paralogues of GILT genes, one of which, GILT3, is expressed in salivary gland and is implicated in slowing the mobility of *Plasmodium* after it is injected into the bloodstream of the human host. The GILT gene expression was measured across different developmental stages in the UCI strain, out of which the larva, pupa and the adult female samples have the highest expression (Fig. 4c). The GILT3 gene in IndCh is structurally disrupted with respect to the UCI strain due to a 20 bp insertion. It is plausible that this disruption is responsible for the higher vectorial capacity of the IndCh strain.

We looked into single nucleotide variation (SNVs), indels and structural variants (SVs) within genes in IndCh compared to the UCI strain. A total of 1.6 million SNVs are found in chromosome 2 of IndCh compared to the UCI genome, of which 19,541 create missense mutations and 3599 fall within the 2R*b* locus. Interestingly, we found 4 missense mutations within the ACE1 protein alone, of which R480L is most likely to impact its function (Fig. 4e). One of the Cyp6a2 transcripts also has a non-consevative, amino acid substitution, A61E, in the IndCh assembly (Fig. 4d). Using the publicly available, developmental transcriptome data, we also analysed the expression patterns of genes within the 2R*b* region that are implicated in insecticide resistance, such as GST and Cyp450 gene clusters. Some of these genes are upregulated in the larval stage in the STE2 strain, implicating them in insecticide resistance (Fig. 4a, b).

There are 3881 SVs within the 2R*b* inversion region in the IndCh assembly compared to the UCI genome, including 1905 deletions, 1727 insertions, 21 copy number variants (CNVs), 185 breakends, 6 inversions, 29 insertion duplications and 8 split duplications. The two coloured heatmap (Fig. 4g) shows the number of deletions and insertions in IndCh compared to chromosome 2 of the UCI assembly (outer blue circle in Fig. 4g), suggesting that the density of SVs within the 2R arm is lesser than that in the 2L arm of UCI. Deletions in IndCh with respect to the UCI genome alone account for 3.8% of the genome (Supplementary Fig. 4). In order to visualise the impact of SVs, it was necessary to liftover the predicted IndCh genes to the UCI genome assembly. The diversity in the two strains posed a major challenge with regards to the liftover. However, this was done using a home-grown tool, which allowed liftover of 80% of the genes, an example of which is shown in Supplementary Fig. 5.

There are 30 SVs that intersect with exons and have the potential to alter the functions of 18 genes within the 2R*b* region, including four genes of unknown function. The 14 functionally annotated genes were checked for presence of more copies, or their paralogues, throughout the genome. Among a few with interesting structural changes are Lin37, MCM3AP, Tigd4, Nop58 and Tango2 genes. The 5’UTR of Lin37 appears to be translocated from the 5’ UTR of MCM3AP in the UCI genome. This region has no repeat element, but is as long as 5 Kbp in size. This translocation has the potential to alter the structure, function or expression of both Lin37 within the 2R*b* region and the MCM3AP gene in chromosome 3. The MCM3AP is a gene involved in the transport of mRNA to the cytoplasm. It acetylates MCM3, which then takes part in DNA replication^24–26^. Lin37 gene is involved in cell-cycle checkpoints and is part of the DREAM complex activated whenever the G1-S phase, cell-cycle checkpoint protein, retinoblastoma, loses function.

Among other structural changes are the first exon of the gene, Tigd4, with a 60 bp deletion in the middle of the first exon in the IndCh assembly (Fig. 4f). The Nop58 gene^27^ in IndCh shows one less exon as compared to that in the UCI genome because of a premature stop codon. Since both Tigd4 and Nop58 have structurally uninterrupted paralogues, CENPB and Nop56, respectively, any negative impact of these deletion/termination on the function may, perhaps, be compensatory. The Tango2 gene in IndCh is a CNV with one copy resulting in a frameshift variant with loss of the start codon. A depletion in the protein product of this gene has been shown to cause fusion of Golgi bodies and the endoplasmic reticulum (ER) in *Drosophila*^28^. Should the Golgi and ER fuse, there will be a metabolic crisis in the cells of the organism. However, in the IndCh assembly, the duplicate copy of the entire Tango2 gene is intact, suggesting active evolution of this gene in IndCh.

The predicted SVs in the IndCh assembly also include large deletions and insertions spanning more than 5 Kbp within the body of the genes, which match with LTR-Gypsy elements in the genome of UCI (Supplementary Table 1). For example, there are 244 deletions larger than 5 Kbp in chromosome 2 of IndCh (black dots against UCI chromosome 2 in Fig. 4g), of which 139 match with the LTR-Gypsy elements in the UCI genome (shown by red dots against the chromosome 2 of UCI in Fig. 4g). In order to show the number of LTR-Gypsy inserted in IndCh, Fig. 4g is complemented with the 2R arm of IndCh, shown as the black outer ring. The insertions that are greater than 5 Kbp in the IndCh strain are marked with green dots and the intersecting LTR-Gypsy elements are shown as orange dots. For example, these LTR-Gypsy elements were also observed to be deleted from the IndCh assembly and IndCh PacBio reads when compared with the UCI genome (Supplementary Fig. 6).

### The 2R*b* inversion signature

**Figure 5:**
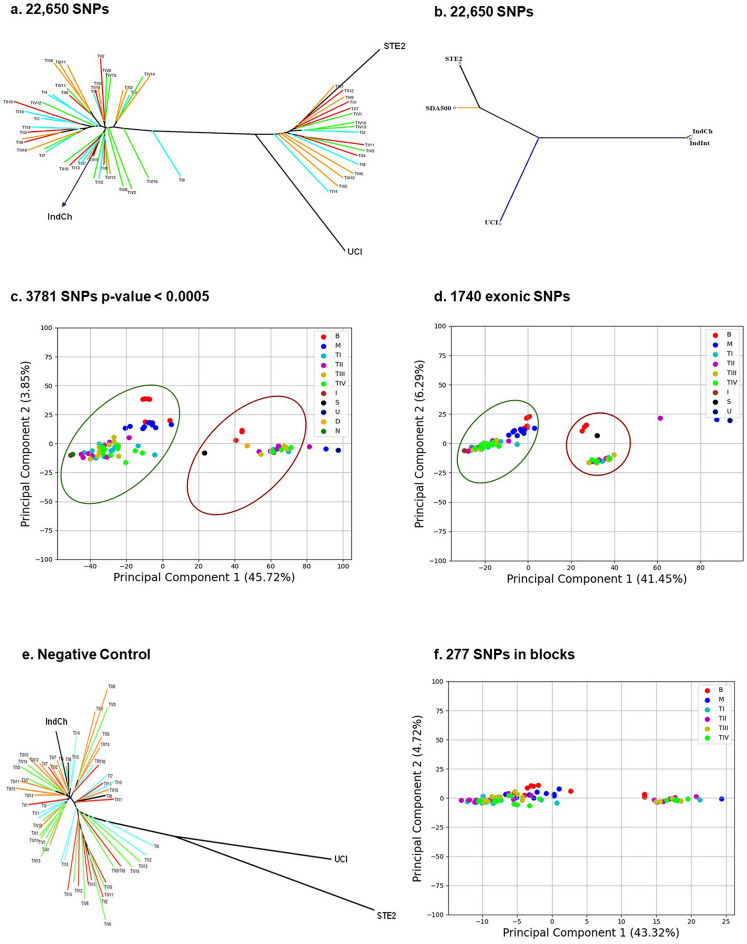
Inversion signature for 2R*b*: (Key : TI—Bangalore Lab; TII—Chennai Lab; TIII—Delhi Lab; TIV—Mangalore Lab; B—Bangalore Wild ; M—Mangalore Wild; I—IndCh ; S—STE2 ; U—UCI; D—SDA500; N—IndInt). (**a**) Phylogenetic tree with 22,650 SNPs for 62 laboratory samples along with the three reference genomes. (**b**) Phylogenetic tree with 22,650 SNPs for five assembled genomes. (**c**) PCA of 62 lab samples and 20 wild samples using 3781 high-significance SNPs (p-value of  < 0.0005) obtained after supervised clustering including genomes of IndCh, STE2, UCI, SDA500, IndInt strains. (**d**) PCA plot of 1740 candidate SNPs found in the exonic regions of the genome. (**e**) Negative control using 17,106 candidate SNPS from chromosome 2L of similar lengths and strategy. (**f**) PCA using 277 SNPs from 31 blocks among the candidate SNPs including wild samples.

**Figure 6:**
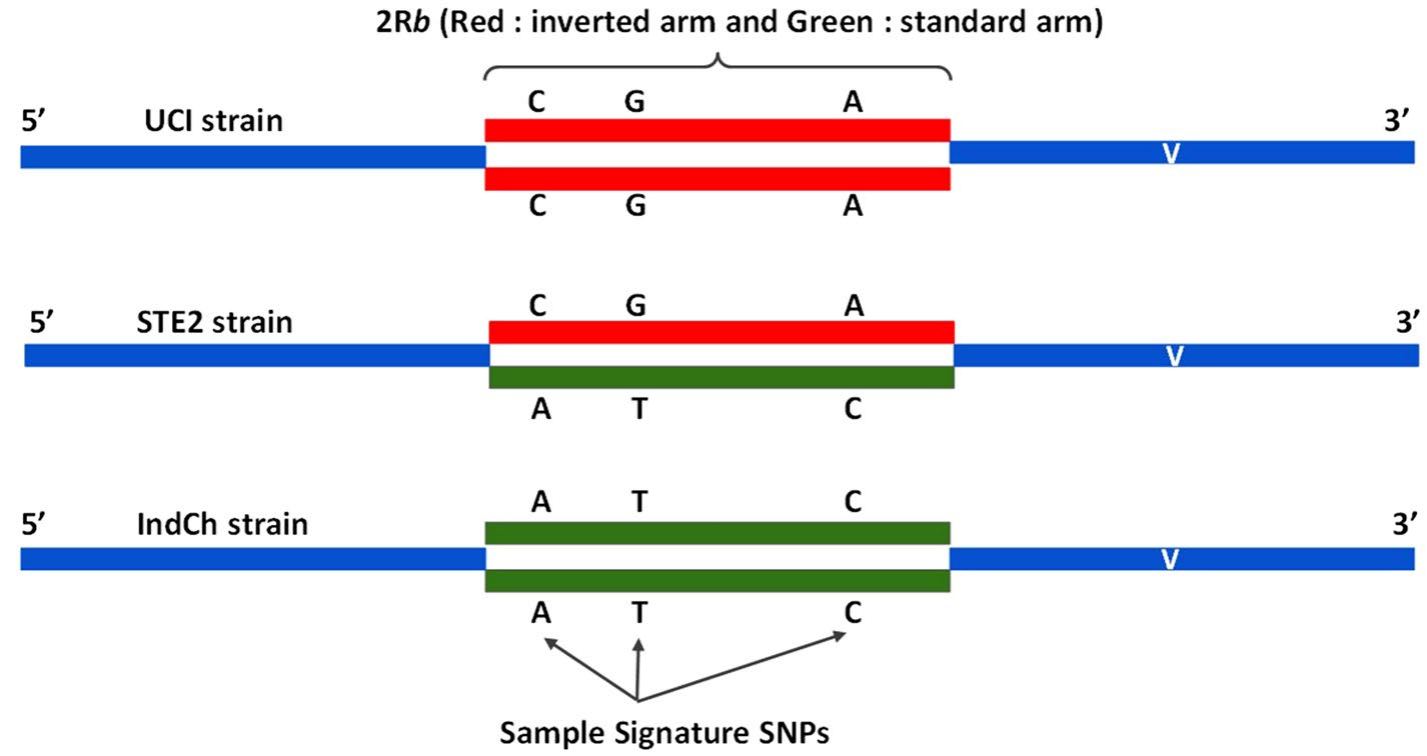
Strategy for selection of SNPs and indels specific for the 2R*b* standard and inverted forms.

The reported genome assembly of IndCh (2R+^b^/2R+^b^) along with the genomes of the other strains STE2 (2R+^b^/2R*b*) and UCI (2R*b*/2R*b*) were used to identify a unique, 2R*b*-specific SNP signature for typing purposes. As shown in Fig. 4h (concentric 5th circle from inside- > out in green), 22,650 SNP positions from within the 2R*b* region that are homozygous alternate alleles in the IndCh assembly, heterozygous alleles in the STE2 genome and homozygous-reference alleles in UCI genome were selected (schematic Fig. 6). This set of SNPs was used to perform unsupervised clustering of 62 individuals from four laboratory reared populations to produce the cluster shown in Fig. 5a. Sixty-five percent (65%) of the individuals are homozygous for the standard form based on the clustering in the same clade with the IndCh strain, and the remaining 35% show the heterozygous configuration for the 2R*b* inversion by clustering in the same clade with the STE2 strain. Figure 5b shows a phylogenetic tree assigning 2R*b* genotype to other strains for which draft genomes are available. Accordingly, the strain SDA500^29^ is heterozygous for the 2R*b* genotype and IndInt strain, an intermediate form of *An. stephensi* for which a genome is currently being assembled in-house (unpublished data), is homozygous for the standard form. As a negative control, a similar strategy was used to extract SNPs from the 2L arm of similar length, which clusters all 62 samples into a single cluster, albeit close to the IndCh genome (Fig. 5e).

The 22,650 SNPs were assigned cluster numbers based on the initial unsupervised clustering from Fig. 5a to perform supervised clustering to extract 3781 SNPs that were most significant with p-values of < 0.0005 (Fig. 4h, concentric 6th circle from inside- > out in blue). The PCA of the 62 individuals from four laboratory reared populations and 20 individuals from two wild populations, using the 3781 SNPs, shows two distinct clusters revealing segregation of individuals by 2R*b* genotype (Fig. 5c) in line with the cluster assignment in Fig. 5a. In order to assign 2R*b* genotypes for individuals directly from transcriptome data, 1740 exonic SNPs were selected out of the 22,650 SNPs, which segregate the 82 individuals into the respective 2R*b* genotypes (Fig. 5d). It should be mentioned that 83% synonymous and 77% of missense mutations from the 1740 exonic SNPs are within genes expressed in publicly available RNA-Seq data across developmental stages for the STE2 strain. This allows assignment of the 2R*b* genotype directly from transcriptome sequencing data to connect the impact of the 2R*b* genotype on gene expression. We have also tried different strategies to reduce the number of signature SNPs to fit validation technologies such as the RFLP-PCR or Amplicon-seq. One such strategy, described in the methods section, allows clustering of individuals by genotype using as low as 277 SNPs as shown in Fig. 5f.

## Discussion

Here, we report the assembly of the genome of a novel strain of *An. stephensi* (IndCh), which was collected in 2016 from Annanagar, Chennai, India and has since been reared in the laboratory. According to this, IndCh displays a homozygous standard form of 2R*b* (2R+^b^/2R+^b^), unlike the genomes of the other two strains STE2 and UCI. The genome assembly of this strain completes the trilogy of configurations with respect to a most frequent inversion 2R*b*. The inversion spans nearly 16.5 Mbp with more than 1300 genes within the 2R*b* inversion region of *An. stephensi* chromosome 2. The complementarity of the IndCh genome with that of the UCI strain with respect to 2R*b* genotype suggests that the thousands of bases inserted at the 2R*b* breakpoints in the inverted form in the UCI strain may help to accommodate loop formation in heterozygous configuration, without disrupting the structures of homologous segments.

In *An. gambiae,* it was reported that transposable elements (TEs), including the LTR-Gypsy elements, accumulate near the 2L*a* inversion breakpoints^30^. In this context, the thousands of bases inserted near the breakpoints in the UCI genome of *An. stephensi*, filled with repeats, suggest that inversions are not just relative arrangement of physical markers, but have bearing in mosquito evolution.

Structural variants caused by repeat elements have been shown to be prevalent among variations of complex traits in *Diptera*^31^. Since retrotransposons, including LTR-Gypsy, impact the expression of genes, insertion and deletion of these elements have been postulated to affect the efficiency of the genes inserted via CRISPR-Cas9 construct in gene-drive technologies^32^. There are a total of 43 predicted genes within the 2R*b* locus in IndCh with LTR-gypsy element inserted/deleted compared to that of the UCI strain, perhaps, regulating their expression differentially depending on 2R*b* genotypes.

**Figure 7:**
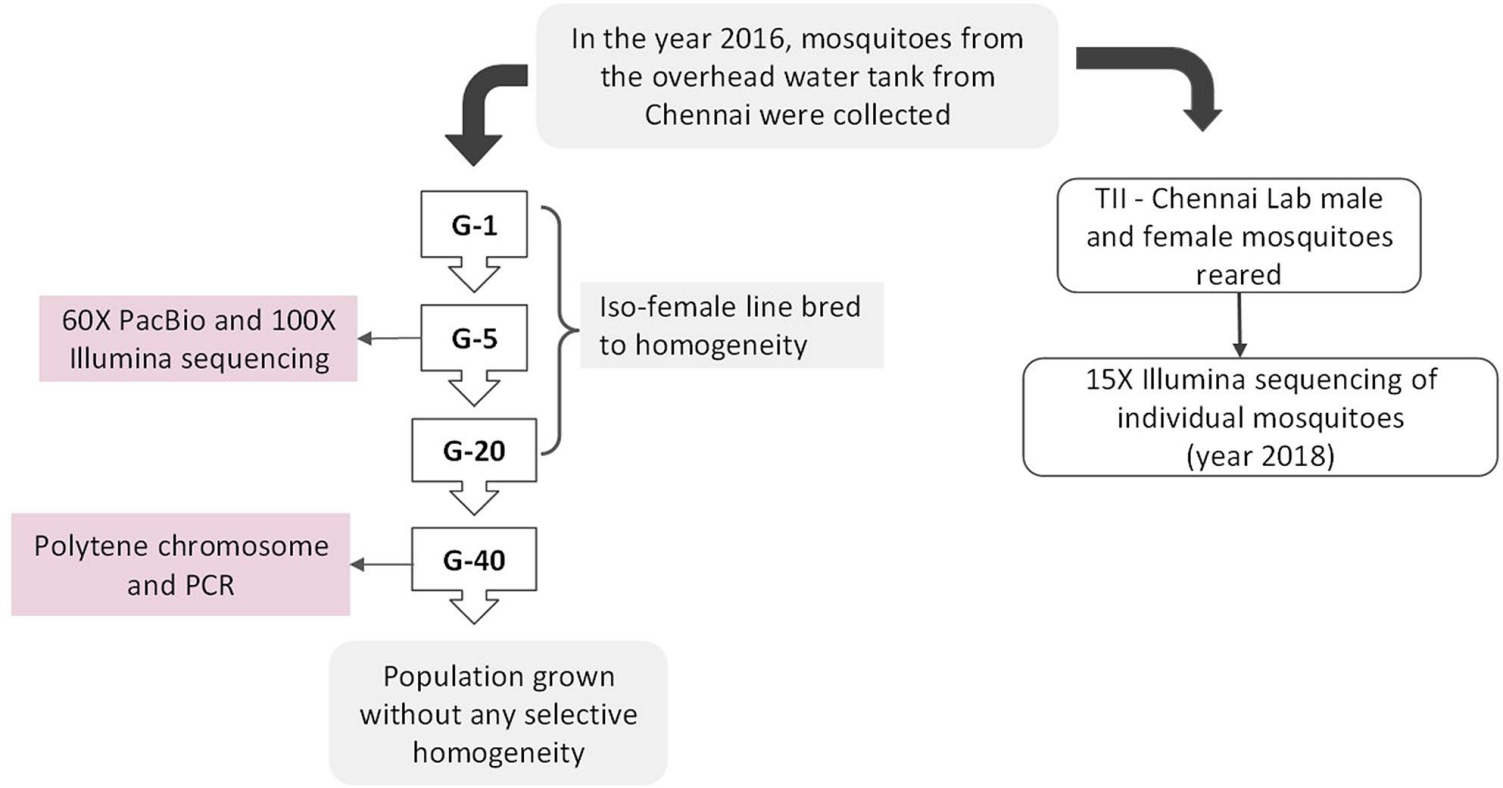
Flow-chart showing the rearing and generation-wise data for Chennai mosquitoes used for raw sequence data, validation using PCR, generating photograph using polytene and analysis.

The unusually large number of segregating SNPs identified allows the opportunity to create several subsets of signatures for typing using diverse cost-effective technologies, such as low-density microarrays, ampli-seq, RNA-seq or PCR. The typing of 82 individuals using WGS demonstrates that 30% of individuals within lab-reared and wild individuals maintain the heterozygous form of the 2R*b* genotype consistent with the observation reported by Mahmood and Sakai^14^. It should be mentioned that even individuals from the original Chennai population, from which the IndCh strain has been homogenised, shows 30% heterozygous forms of 2R*b* (Fig. 5a). The homozygosity achieved for IndCh strain is evident from the remnant very low (3.18%) heterozygosity even in the outgrown iso-female line, maintained separately over 35 more generations (see Fig. 7 and Supplementary Fig. 7).

A comparison of genome-wide variants among the genomes of the three strains of *An. stephensi* reveals that the IndCh genome has ~2 million variants against the UCI strain, which is 3-fold more than the ~600K variants seen between the STE2 and the UCI genomes. Furthermore, in IndCh, there are 30K missense mutations within the 14K predicted genes reported for the UCI genome^9^ compared to only 10K missense mutations in STE2, in comparison to the UCI strain. These observations along with the clustering of the 82 individuals from the four geographically diverse populations in the same clade with IndCh (Fig. 5e) would suggest that IndCh has not only diverged from the other two strains, but significant cross breeding may have happened in recent times with increased commerce and travel between major cities.

According to Lukyanchikova et al*.* 2020^8^, in AsteI_V4 assembly of the strain STE2, the distal 2R*b* breakpoint is reported at 21.14 Mbp and proximal breakpoint is at 37.18 Mbp. In IndCh, both the distal and proximal breakpoint coordinates are shifted by ~ 0.37 Mbp and ~ 0.90 Mbp, respectively, towards the centromere. This could be because the draft scaffolds used to assemble the genome of the STE2 strain were generated using low coverage PacBio RS1 technology back in 2014 and scaffolded using mate-pair sequences before scaffolding with the HiC data. Also, AsteI_V4 is assembled from sequencing polymorphic individuals from the lab strain, which can pose considerable challenge in the assembly near the breakpoints. On the other hand, both IndCh and UCI assemblies were generated independently using iso-female lines homogenised over multiple generations. We have aligned the 2R arm of AsteI_V4 and UCI against IndCh assembly using minimap, helping to study the shift in the breakpoint coordinates away from the centromere in the IndCh genome (JBrowse link for IndCh with the track name ‘sorted_IndCh2R_AsteiV4_Chr2R’). It should be mentioned that the size of the 2R*b* region in IndCh assembly compares with that of the UCI assembly.

## Conclusion

To our knowledge, this is the first instance where high-resolution genomes have been independently assembled for multiple strains representing all three genotypes of a given inversion in *Anopheles* species. More specifically, the high-resolution assembly of the genomes of the two strains, UCI and IndCh, that are homozygous to inverted and standard forms of 2R*b*, respectively, allowed base-level characterisation of breakpoints with high confidence. Also, this has allowed identification of 22,650 segregating SNPs using comparative genomics and, for the first time, one can type the 2R*b* inversion *in An. stephensi directly* using transcriptome sequencing, a critical step in the identification of candidate genes within this inversion region that may be responsible for traits like insecticide resistance, circadian rhythm and adaptation to climate. We believe that the IndCh genome reported here is a more relevant reference for malaria management in urban settings within India because there is significant divergence in IndCh from the earlier strains. The genome trilogy with respect to the 2R*b* genotype reported here and findings from comparative genomics have placed the genome resource of *An. stephensi*, a grossly understudied malaria vector until recently, on par with the genome resources accumulated over two decades for the most extensively-studied malaria vector, *An. gambiae*.

## Methods

### Collection and maintenance of mosquito colony

*An. stephensi* was collected from Annanagar, Chennai, India and is being maintained in the insectarium of Tata Institute for Genetics and Society (TIGS, India). For routine maintenance, larvae are given food prepared by mixing Brewer’s yeast powder and dog food (Pedigree brand chicken and vegetable mix) at a ratio of 30:70^34^. Pupae are bleached with 1% sodium hypochlorite for 1 min^33^ and kept in a mosquito-rearing cage (Bugdorm-4S3030) (W32.5 × D32.5 × H32.5 cm) for eclosion. The emerging adults are fed on a mixture solution of 8% sucrose and 2% glucose mixed with 5% multivitamin syrup (Polybion LC®)^34^. A standard membrane feeding system with modification was followed as described in Gunathilaka et al*.*^35^*.* On the 3^rd^ day, eggs are collected and allowed to hatch into larvae in rearing trays (Polylab, Catalogue no. 81702) (L39 x B30 cm). The larval stage of this mosquito is completed within 12–14 days. Adults are maintained in the insectarium at 28 ± 1 °C temperature and relative humidity 75 ± 5% with a photoperiod of 12:12 h dark and light cycles.

Biosafety approval for mosquito maintenance facility (approval Ref. No.TIGS 2nd IBSC Oct 2018) and Institutional ethical approval for use of human blood for mosquito feeding were obtained (approval Ref. No. inStem/IEC-12/002).

In this study the human data/samples have not been used directly but have been sourced through a licensed blood bank. The blood used during feeding mosquitoes for colony maintenance was collected from a licensed human blood bank (Lion’s Blood Bank, Bangalore). The human ethical approval has been cleared vide Ref No. inStem/IEC-12/002 from Institute for Stem Cell Science and Regenerative Medicine (inStem).

### Establishment of iso-female lines

The IndCh iso-female line of *An. stephensi* was generated from the above-mentioned mosquito colony following the protocol of Ghosh & Shetty^36^. Blood meal was given to 5-day old adult females. Twenty-five gravid females were selected and kept separately. On day 3, each gravid female was separated in small cups lined with filter paper and filled with ¼ water. Each cup was covered with a mosquito net and labelled. Cotton balls soaked with sugar solution were kept on top of each cup. Females from five founder lines that laid greater number of eggs and had 90–95% hatchability were selected. These selected lines were maintained separately to generate iso-female lines. The larvae and adults were given the same food as mentioned earlier. Adults emerging from each line were kept separately in mosquito rearing cages (Bugdorm-4S1515) (W17.5 × D17.5 × H17.5 cm) and allowed for sibling mating. On day 5, adult females of each line were blood fed and gravid females were separated in cups to obtain the next progeny in the similar way as mentioned above. The same procedure was continued for 5 generations to establish a homozygous IndCh iso-female line for sequencing.

### Karyotyping of polytene chromosome

Polytene chromosomes were prepared from the ovarian nurse cells collected from semi-gravid females of the IndCh iso-female line in the 40th generation as per the method of Ghosh & Shetty^37^. The semi-gravid females were anaesthetised and placed on a microslide in a drop of diluted Carnoy’s fixative solution (Carnoy’s fixative: distilled water, 1:19). The ovaries were pulled out and fixed in modified Carnoy’s fixative (methanol: acetic acid, 3:1) for 2–3 min. After fixation, the ovaries were stained with lacto-acetic orcein for 15–20 min. After staining, 60% acetic acid was added and a clean coverslip was placed on the top of the stained sample. Gentle pressure was applied on the cover glass for squashing. The edges of the coverslip were sealed with nail polish. The slides were examined under microscope at 400X and 1000X, respectively, for inversions. The inversion nomenclature and their frequency were recorded^5^.

### Assembly

PacBio reads from the IndCh strain were assembled independently using Canu^20^ and FALCON^23^ assemblers. The resulting assemblies were combined using Quickmerge^21^, where the Canu assembly was taken as reference and the FALCON assembly served as the query. Two rounds of Arrow polishing (PacBio gcpp v2.0.2) using PacBio reads, followed by two rounds of Pilon polishing (v1.22)^22^ using 100 × Illumina reads, were carried out on the merged assembly. HiC data from the STE2 strain of *An. stephensi from the public databases* was utilised to scaffold the contigs using SALSA^38,39^ by mapping the HiC reads to the contigs using the Arima Genomics mapping pipeline. DNA physical marker sequences for each chromosome arm published by Jiang et al*.*^6^ were downloaded and aligned against the scaffolds using BLAST^40^, in order to assign the chromosomal positions to the scaffolds. The scaffolds were stitched based on the order and orientation of the markers into pseudo-chromosomes. Hundred (100) N’s were added between two scaffolds during stitching. Physical marker sequences were realigned to the stitched chromosomes for validation. Coordinates from the BLAST output, along with the stitched chromosome lengths, were utilised to generate a karyogram for visualisation.

### Haplotype phasing

The assemblers FALCON and FALCON-Unzip were used to phase the IndCh genome into haplotypes. Raw IndCh PacBio reads were used by the FALCON assembler to produce a set of primary contig files and an associate contig file representing the divergent allelic variants. The output of primary and associate contig files were then used by FALCON-Unzip to produce partially phased primary contigs (all_p_ctg.fa) and fully phased haplotigs (all_h_ctg.fa), which represent divergent haplotypes. Polishing was performed on the phased contigs by FALCON-Unzip using Arrow.

### Purge haplotigs

The tool ‘Purge Haplotigs’^41^ was used to determine the degree of heterozygosity in the IndCh strain. PacBio reads of IndCh strain were mapped to the consensus primary assembly (cns_p_ctg.fasta) obtained from FALCON-Unzip to obtain an aligned BAM file. Coverage analysis was performed on the BAM file to generate a read-depth histogram.

### Identification of 2R*b* Breakpoints in the UCI and IndCh strains

The tools HiC-Pro and Juicebox were used to generate a contact map for a strain by mapping the HiC reads from one strain onto the genome with different inversion genotype to produce a butterfly-like structure representing the breakpoints, which were visualised and extrapolated from the contact map of the tool Juicebox. The breakpoints were validated by mapping the PacBio and Illumina reads onto the respective genomes. Alignment using ‘unimap’ and BLAST was performed between the genomes of the IndCh and UCI strains, to determine the precise breakpoints for the 2R*b* inversion to consolidate with the findings from HiC-Pro.

### Repeat analysis and annotation

A database for a given assembly was created using the ‘BuildDatabase’ utility. This database was submitted to the RepeatModeler version 2.0.1^42^ along with the -LTRStruct parameter to predict the long terminal repeats. The custom library of consensus sequences < database_name > -families.fa from the RepeatModeler output and the -no_is parameter for skipping bacterial insertion elements were given as input for masking the repetitive elements using RepeatMasker version 4.1.0^43^.

### Insect samples

In total, 82 mosquito samples, which include 4 lab strains TI Bangalore Lab (n = 15), TII Chennai lab (n = 15), TIII Delhi Lab (n = 16), TIV Mangalore Lab (n = 16) and 2 wild-type strains from different geographical regions of India (Bangalore, n = 10 and Mangalore, n = 10), were used in the present study. Lab strains were obtained courtesy of Dr. S. K. Ghosh (NIMR, Bangalore).

### Isolation of genomic DNA

Briefly, the insect tissue was homogenised and the genomic DNA was isolated using Qiagen Genomic-tip (Qiagen). Later, fluorometric quantification of DNA was done using Qubit (Invitrogen). This procedure was adapted from our recent publication^3^.

### Library preparation and sequencing

Whole Genome DNA libraries with an average insert size of 200 bp were made using NEBNext® Ultra™ II DNA Library Prep Kit for Illumina® (New England Biolabs, 2016) using the protocol recommended by the company. Briefly, around 50 ng of DNA was used for library preparation, DNA was sheared using Adaptive Focused Acoustic technology (Covaris, Inc.) to generate fragments of length around 200 bp. The fragments were end repaired, 3′-adenylated, ligated with Illumina adapters, and PCR enriched with Illumina sequencing indexes. The size selection was performed using solid-phase reversible immobilisation (SPRI) beads (Agencourt AMPure XP Beads) from Beckman Coulter. The quality and quantity of the libraries were evaluated using Qubit (Invitrogen) and TapeStation (Agilent). The libraries were diluted and pooled with an equimolar concentration of each library. Cluster generation was done using cBot (Illumina) and paired-end sequenced on Illumina HiSeq 2500 platform using TruSeq SBS Kit v3-HS (200 cycle) (Illumina, San Diego, CA) following the manufacturer’s recommendations. This procedure was adapted from our recent publication^3^.

### Gene annotation

AUGUSTUS (version 3.2.3)^44^, an eukaryotic gene prediction tool, was used to find protein-coding genes for all the assembled genomes. The model organism closest to *An. stephensi, Ae. aegypti,* which was made available by AUGUSTUS, was used for gene prediction. The gff files from AUGUSTUS are uploaded to the browser, the link to which can be found in the section “Data Availability Section”. These gff files were used for gene liftover between different assemblies. This procedure was adapted from our recent publication^3^.

### Liftover

The two genomes were aligned using nucmer (NUCleotide MUMmer). Parameters ‘–maxmatch’ and ‘–noextend’ made sure to show all alignments regardless of their uniqueness, and to not extend clusters of alignments to make a longer alignment respectively so as to be able to detect structural variants. The delta file obtained from the nucmer run was converted to a text file containing coordinates of the alignment blocks between the two assemblies using the show-coords utility. This output file was converted to a bed file format, which contained the start and stop coordinate information of alignment blocks from both the assemblies and was stored along with the strand direction in the fourth column. The bed file can be viewed on the genome browser provided here under the “Data Availability and Methods” section along with the reference (UCI) to view genome liftover with a trackname titled ‘IndCh_UCI_BED’.

This genome liftover was then used as a base to lift the predicted genes of the assembly reported here to the coordinates of the predicted genes from the reference genome. For each gene, an alignment block was chosen, which are from the same chromosomes, and which encompasses the start and stop codon of the gene/feature (exon or CDS). Based on the strandedness in the alignment block and the strandedness of the gene, the corresponding start and stop coordinates for the reference genome were determined. This can be viewed on the genome browser as the track titled ‘IndCh_Gene’.

### Structural Variant Identification

PacBio structural variant calling and analysis tools, collectively called as PBSV (https://github.com/PacificBiosciences/pbsv) were used to discover structural variants in both the assemblies to be compared. The VCF file from PBSV was used to analyse different structural variants like deletion, insertions, CNVs etc. from the ‘INFO’ column by filtering the ‘SVTYPE’.

### Structural variant annotation

SnpEff was used to annotate SVs and predict their effect on known genes. A snpEff database was created using the reference genome and the gff file obtained from MAKER^9^ annotation. This was then used for functional annotation of the SVs created using PBSV.

### PCR validation

Primers were designed to validate the 2R*b* breakpoints from the IndCh assembly using the tool ‘Primer-BLAST’. The forward primer for IndCh proximal breakpoint (5′ GGGGATGGGAACGTGTTTCATA 3′) and the reverse primer sequence (5′ CATTCGCCACGTTTCAACTCAC 3′) was used to amplify the region of IndCh chromosome 2R from 38,082,572 to 38,083,196 bp. Similarly, the forward primer for IndCh distal breakpoint (5′ TCGTCCCATTTCAGTCGGTAG 3′) and the reverse primer sequence (5′ ATGGTTGCTAAGCACGATGCAG 3′) was used to amplify the region of IndCh chromosome 2R from 21,513,342 to 21,514,478 bp.

The DNA of individual mosquitoes were isolated from IndCh iso-female sibling colony and TII chennai lab colony using Qiagen Blood and Tissue Midi kit (Cat. No. 69506). The fluorometric quantification of DNA was done using NanoDrop (Thermo Scientific 2000 Spectrophotometers).

Each PCR reaction mixture (20 µl) contained 1 µl gDNA, 50 ng each forward and reverse primers (as mentioned above), 10 µl of Q5® Hot Start High-Fidelity 2X Master Mix and 8 µl of Nuclease free water added. A hot start of 95 °C for 2 min, followed by 0.40 min denaturation, 70C annealing and 30 cycles of extension at 72 °C was performed. The PCR products were cleaned using a QIAquick PCR Purification Kit and given for Sanger sequencing.

### 2R*b* inversion signature

Illumina reads for 15X coverage were generated for 82 samples, belonging to 2 wild and 4 lab populations. These reads were mapped to the high-quality genome of the reference using bowtie2^45^. Variants were called using Samtools and the filtration was done on the resulting VCF files using bcftools^46^.

Fastq files of Illumina reads for a coverage of about 100X from the UCI (SRR11672504) and the STE2 (SRR1168951) strains were sourced from NCBI SRA (https://www.ncbi.nlm.nih.gov/sra). This along with 100X coverage of the reported strain were mapped against the high-quality genome of UCI. The resulting VCF files served as the training dataset for inversion signature. The strategy for selecting the 22,650 signature SNPs is shown in Fig. 6.

The alleles at the 22,650 positions were called from 82 individuals from 6 populations to attempt unsupervised clustering using neighbour joining tree. A supervised clustering was performed using DESeq under the Bioconductor package of R, after cluster assignment for each of the 82 samples from the previous step to identify 3781 most significant SNPs with a p-value of < 0.0005. The number of SNPs were further reduced to 277 by selecting only those SNPs from the 3781 which fall in clusters of more than 6 within a stretch of 1500 bases. Also, we identified 1740 SNPs within exons from the 22,650 SNPs to enable 2R*b* genotyping directly from transcriptome data.

## Data availability

The raw PacBio/ Illumina reads used in the assembly and the individual WGS data used to validate 2R*b* signatures are uploaded to the NCBI server under the BioProject ID: PRJNA746765. Click here to Download 22,650 SNPs, 3781 SNPs, and 1740 SNPs signatures. For a browser with UCI Genome as reference click here JBrowse (see Supplementary Table 2 for track information). For the browser using IndCh Genome as reference click here JBrowse (See Supplementary Table 3 for track information).

The flow-chart below shows the source of IndCh strain, which is derived from Chennai lab strain collected from an inner urban setting at a construction site at Annanagar, Chennai in 2016 with GPS coordinates in Supplementary Table 4, by establishing a iso-female line to homogenise the population over 5 generations. The iso-female line was continued until 20 generations and left to sustain as a separate lab colony for another 20 generations. All validation reported here including PCR and polytene chromosome work is done using the outgrown population and the original Chennai-lab strain (Fig. 7).

### Public source of data used in this report

Fastq files of Illumina reads for a coverage of about 100X from UCI strain was downloaded from accession SRR11672504 and STE2 strain from accession SRR1168951. Developmental transcriptome data were obtained from the SRA accession SRP013839. Scripts used for analysis can be obtained from Github. Additional Information related to genes of interest and other raw data can be obtained from the browser IndCh website. The UCI HiC data was accessed from BioProject ID PRJNA629843 and the STE2 HiC data was obtained from a public source (NCBI SRA : SRR11508457).

## Supplementary Information

Supplementary Information.

## Abbreviations

IndCh: India, Chennai strain
SNP: Single Nucleotide polymorphism
PCR: Polymerase Chain Reaction
LTR: Long Terminal Repeats
UCI: University of California, Irvine
HiC: High throughput chromatin conformation capture
NIMR: National Institute of Malaria Research
DNA: Deoxyribonucleic Acid
WHO: World Health Organization
RFLP: Restriction Fragment Length Polymorphism
BLAST: Basic Local Alignment Search Tool
SNV: Single Nucleotide Variant
CNV: Copy Number Variants
UTR: Untranslated Region
PCA: Principal Component Analysis
BAM: Binary Alignment Map
GFF: General Feature Format
CDS: Coding Sequences
PBSV: PacBio Structural Variant
VCF: Variant Call Format
GPS: Global Positioning System
WGS: Whole Genome Sequencing
