# Supplementary material for: The genome trilogy of Anopheles stephensi, an urban malaria vector, reveals structure of a locus associated with adaptation to environmental heterogeneity
Source: Sci Rep. 2022 Mar 4;12:3610. doi: 10.1038/s41598-022-07462-3 (PMC8897464; doi:10.1038/s41598-022-07462-3)
Supplement: Supplementary file 1 — Supplementary Information. [file 41598_2022_7462_MOESM1_ESM.pdf]

The genome trilogy of *Anopheles stephensi*, an urban malaria vector, reveals structure of a locus associated with adaptation to environmental heterogeneity

Aditi Thakare[1,!], Chaitali Ghosh[2,!], Tejashwini Alalamath[1,!], Naveen Kumar[2], Himani Narang[1], Saurabh Whadgar[1], Kiran Paul[1], Shweta Shrotri[1], Sampath Kumar[2], Soumya M[2], Raksha Rao[1], Mahul Chakraborty[3], Bibha Choudhary[1], Susanta K. Ghosh[5], Suresh Subramani[2,4], Sunita Swain[2*] and Subhashini Srinivasan[1,2,*]

[1]Institute of Bioinformatics and Applied Biotechnology, Biotech Park, Electronic City Phase I, Bengaluru 560100, India

[2]Tata Institute for Genetics and Society, Center at inStem – GKVK campus, Bellary Road, Bangalore 560065, India

[3]University of California, Irvine, CA 92697, USA

[4]University of California San Diego, La Jolla, CA 92093, USA

[5]National Institute of Malaria Research, Bangalore 562110, India

* sunita.swain@tigs.res.in and ssubha@ibab.ac.in (Corresponding Authors)

! authors have contributed equally

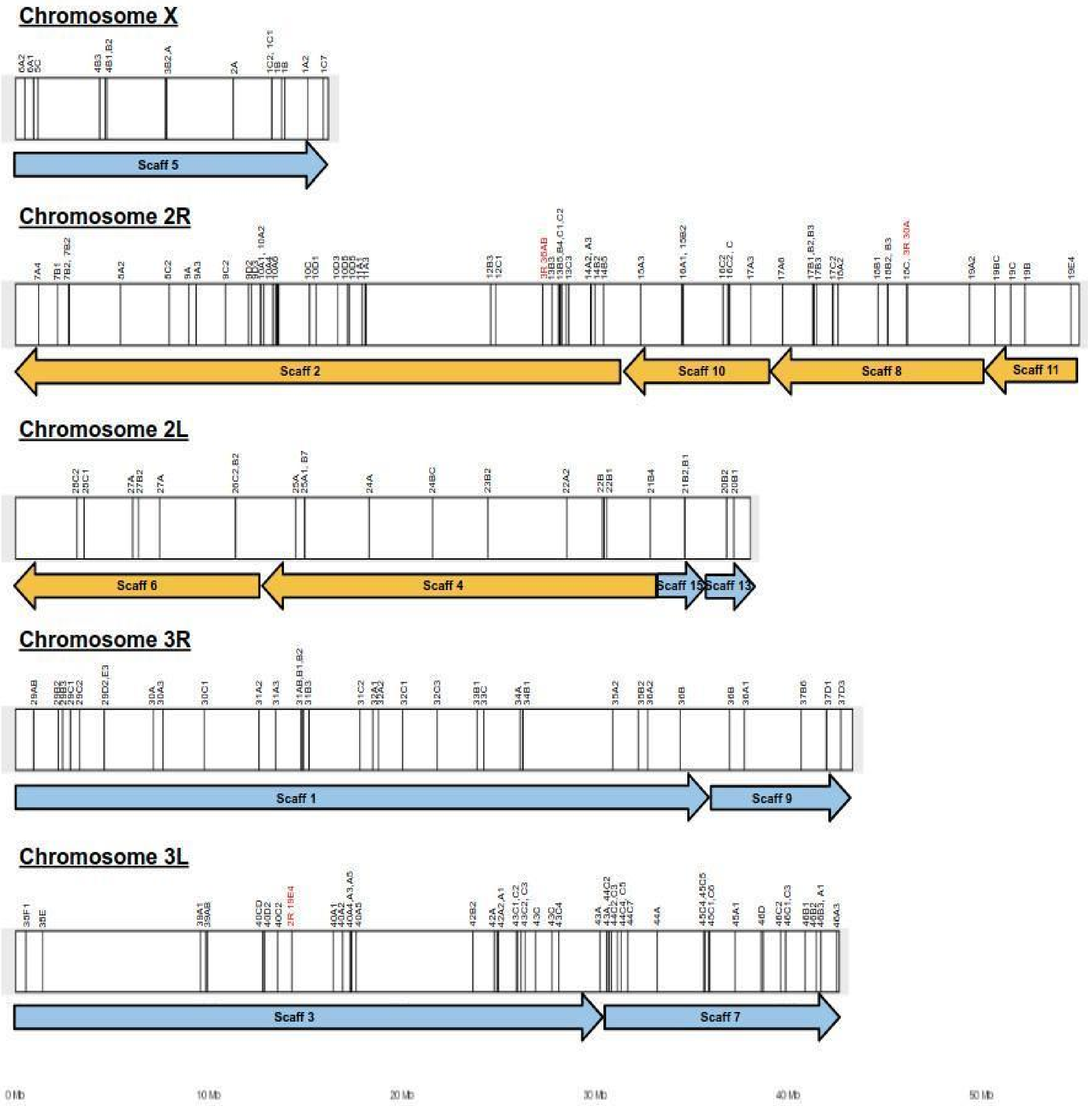

Supplementary Fig. 1: Karyogram of scaffolds from HiC-based assembly stitched using physical markers for IndCh.

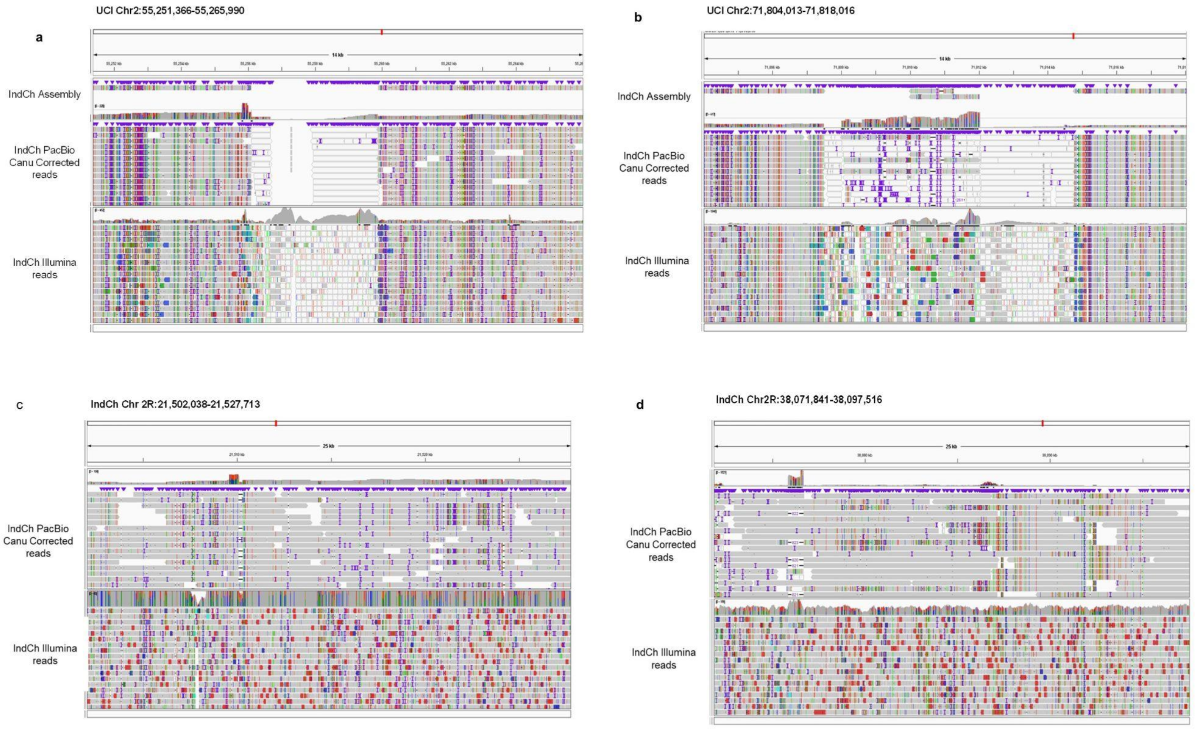

Supplementary Fig. 2: Integrated Genome Viewer (IGV) visualization of CANU corrected IndCh PacBio reads (top) and IndCh Illumina reads (bottom) mapped on (a) Proximal breakpoint of UCI assembly (b) Distal breakpoint of UCI Assembly (c) Distal breakpoint of IndCh assembly (d) Proximal breakpoint of IndCh assembly.

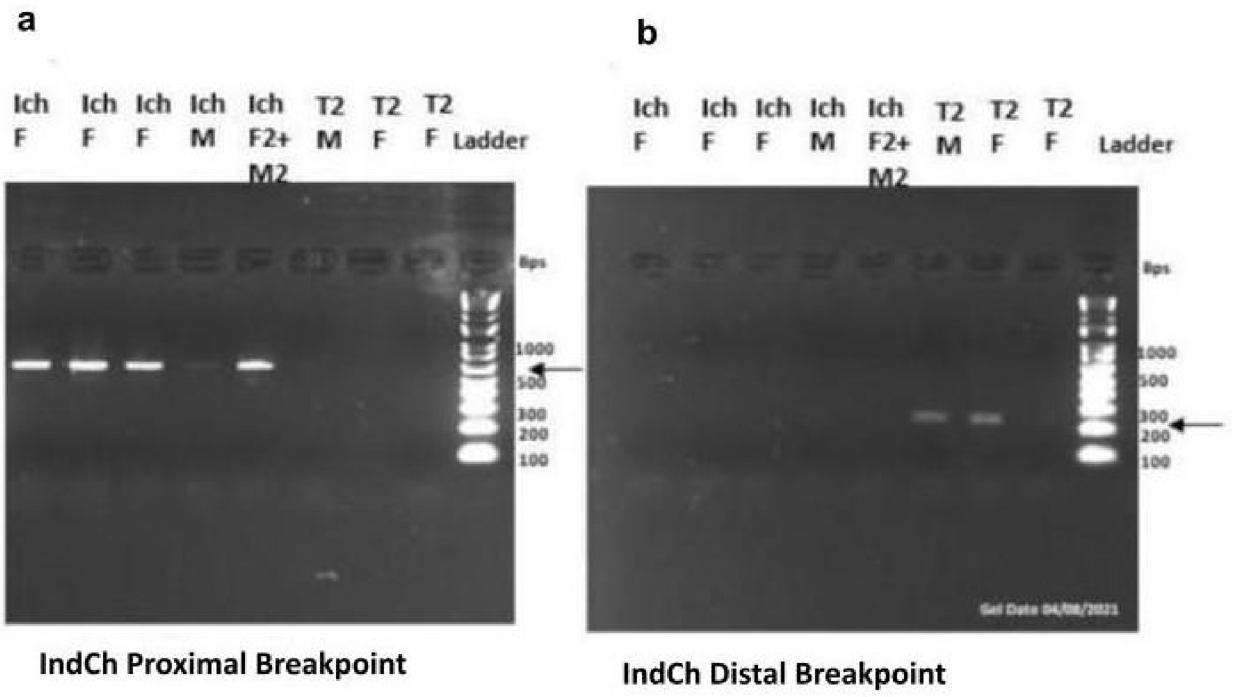

Supplementary Fig. 3: Agarose gel stained with SYBR Safe DNA gel stain. The amplicon size was determined by comparing it with Invitrogen 1kb Plus DNA Ladder. (a) The proximal breakpoint from the IndCh assembly amplified in outgrown IndCh (Ich) population and from original Chennai lab population (T2) from which IndCh strain was derived (b) The distal breakpoint from the IndCh assembly amplified only from Chennai lab population (T2). F is 'Individual Female Mosquito', M is 'Individual Male Mosquito', F2+M2 is two female mosquitoes and two male mosquitoes.

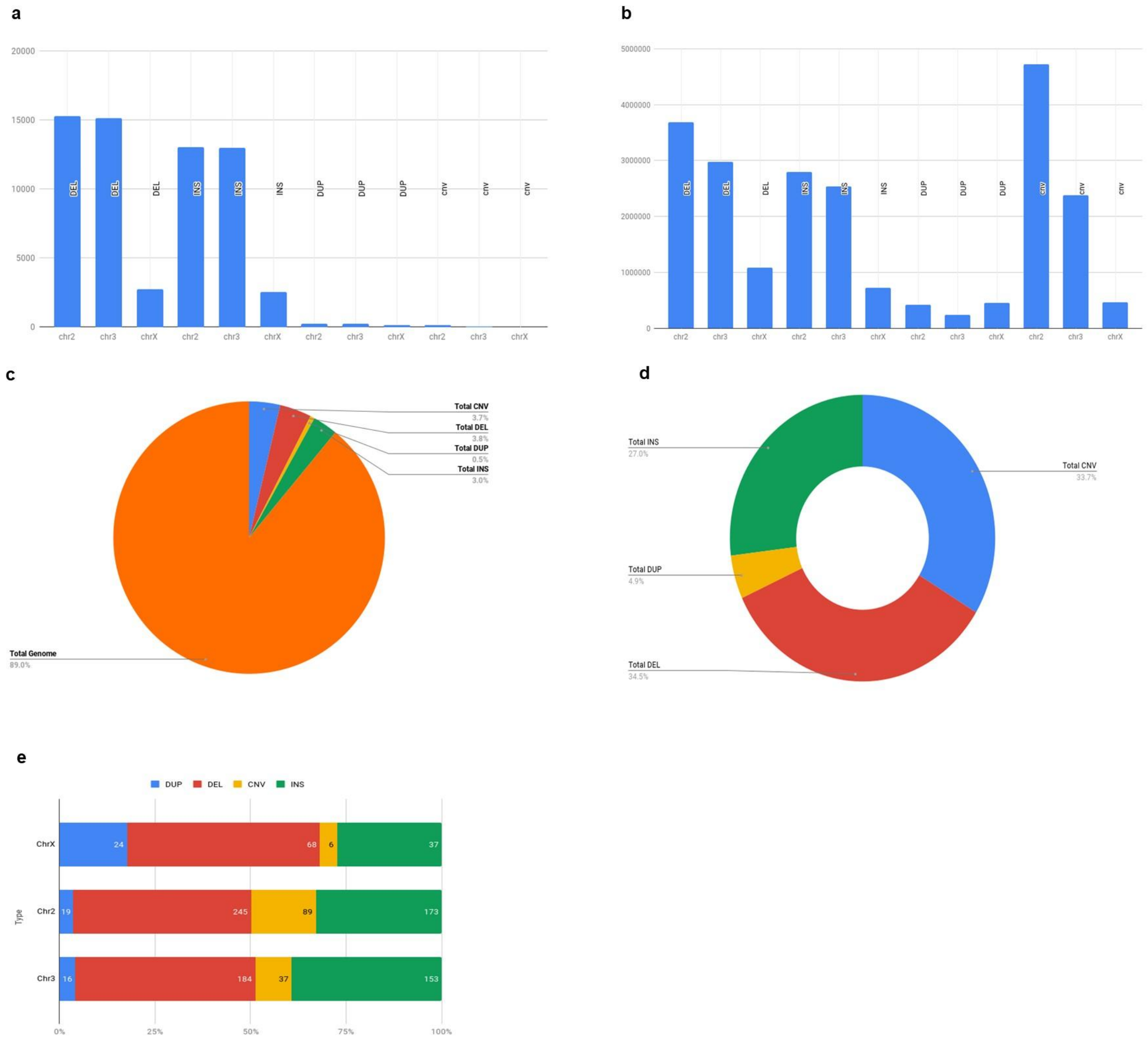

Supplementary Fig. 4: (a) Shows number of structural variants present per chromosome, (b) shows the number of base pairs involved in each type of SVs per chromosome, (c) shows percent base pairs from the whole genome (chr2, chr3 and chrX) implicated in SVs. CNV and DEL accounts for almost 6% of the genome, (d) shows relative abundance of SV, DEL, INS, DUP and CNV, (e) shows relative distribution of SVs higher than 5 Kbp length.

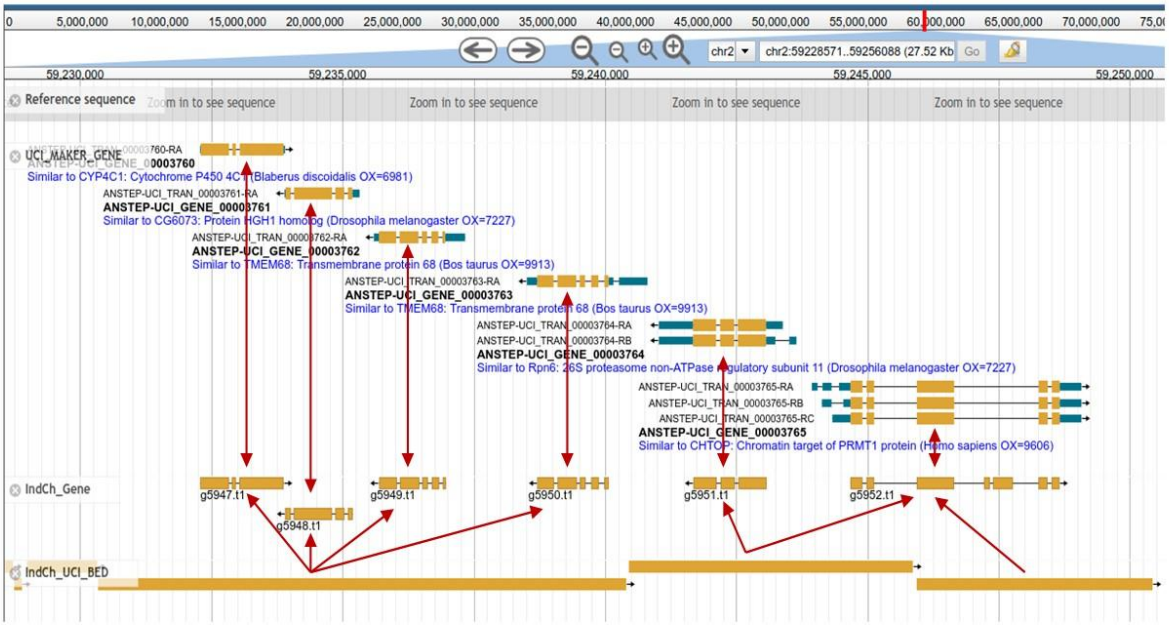

Supplementary Fig. 5: A snapshot of the genome browser showing genome and gene liftover. The first track, "Reference sequence", is the UCI sequence. The next track, "UCI_MAKER_GENE", shows the Maker predicted genes for the UCI strain. The track "IndCh_Gene" shows the IndCh Augustus predicted genes lifted over to UCI coordinates. The track "IndCh_UCI_BED" shows the alignment blocks between IndCh and UCI on UCI coordinates. The six UCI genes shown here within the locus chr2:59,228,566-59,249,465 of UCI genome have corresponding genes in IndCh (marked with double headed arrows). Four alignment blocks used to lift over the genes are also shown.

\

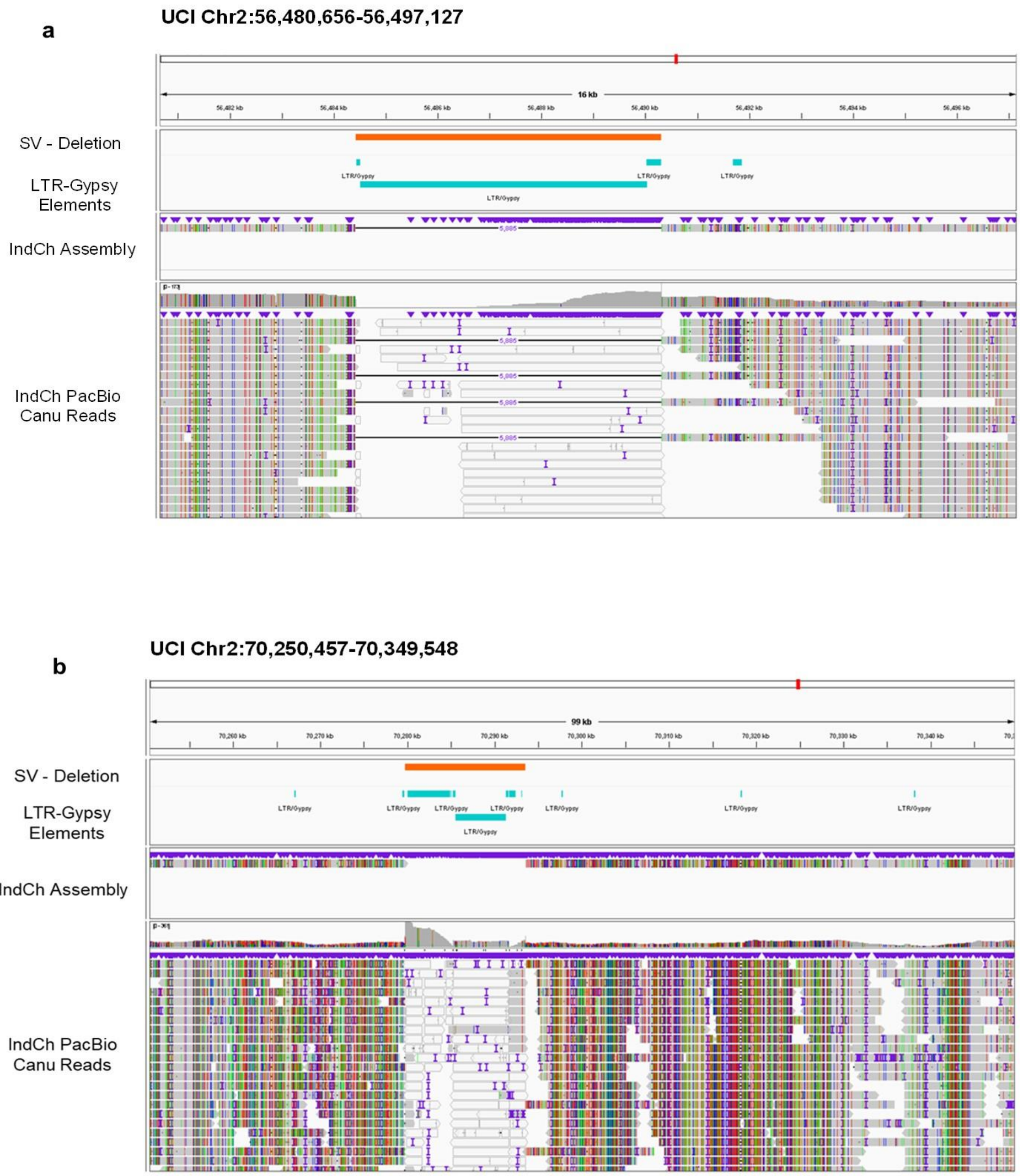

Supplementary Fig. 6: Two examples (a and b) of IGV visualization supporting two LTR-Gypsy elements deleted from the IndCh assembly (top) and IndCh PacBio reads (bottom) when compared against the UCI genome.

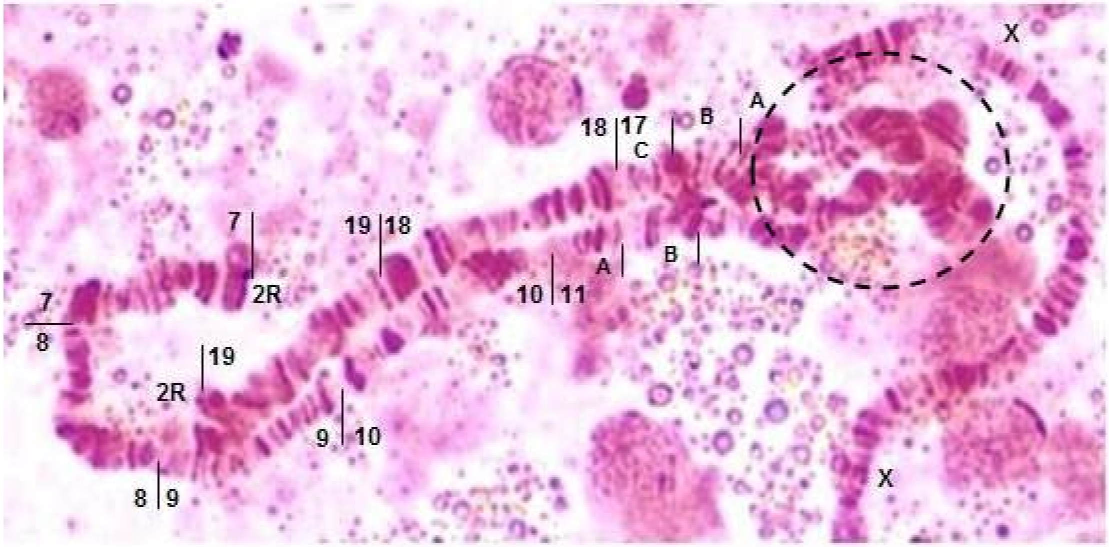

Supplementary Fig. 7: Photograph supporting heterozygous 2Rb inversion in an individual. The black dotted circle shows the loop formation exhibiting the heterozygous form in 3.18% of the IndCh outgrown iso-female line.

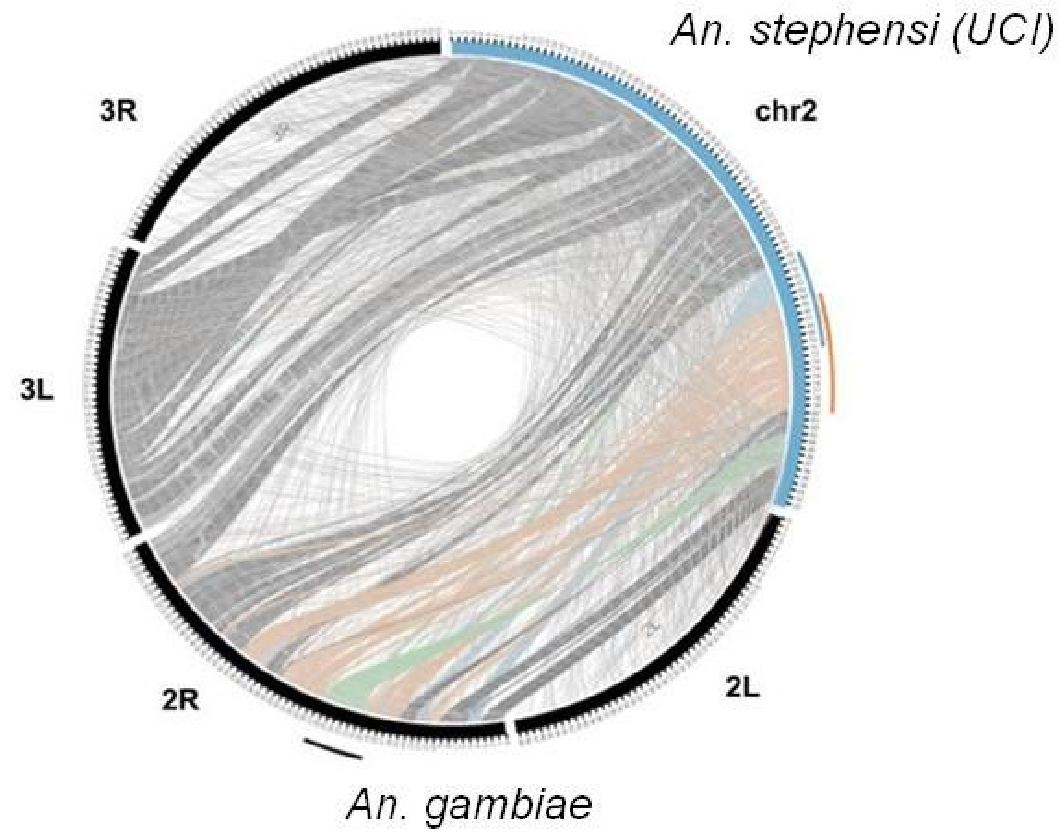

Supplementary Fig. 8: Comparison between An.stephensi and An.gambiae genomes, showing no synteny within the 2Rb region. Blue arc represents the 2Rb inversion region in An. stephensi, orange arc is for the 2Ri inversion An. stephensi. Black arc shows the 2Rb inversion of An. gambiae. The colored connections highlight synteny.

**From PBSV**	**Chr X**	**Chr 2**	**2R*b***	**Chr 3**	**Genome -wide**	**Genome-wide validation from PBSV with SVMU**
No. of deletions/insertions (>= 5 Kbp) in IndCh	63/20	244/151	56/28	183/101	490/272	445/248
No. of deletions/insertions (>= 5 Kbp) in IndCh intersecting with the LTR-Gypsy elements (>= 1 Kbp)	32/12	139/58	31/10	99/31	270/101	249/94
No. of LTR-Gypsy (> 1 Kbp) elements spanning the deletions/insertions in IndCh	47/12	162/43	34/11	117/28	326/83	297/105
No. of the intersected LTR-Gypsy elements spanning in UCI genes (purple) / IndCh genes (brown)	36/6	159/25	38/5	105/19	300/50	NA
No. of reported LTR-Gypsy elements in UCI (>= 1 Kbp)	413	347	50	209	969	NA
No. of predicted LTR-Gypsy elements in IndCh (>= 1 Kbp)	24	112	24	93	229	NA

Supplementary Table 1: Stats showing deletions and insertions from PBSV of more than 5 Kbp in length observed in IndCh and their intersection with the LTR-Gypsy elements of length more than 1 Kbp. The last column validates the deletions and insertions from PBSV with SVMU.

No.	File Type	Track Name	Track Content
1	FASTA	Reference Sequence	*Anopheles stephensi* genome 2.0 (UCI)
2	GFF	Augustus	Augustus predicted gene for UCI reference genome
		IndCh Gene	IndCh-UCI Lifted Over genes
		UCI Maker Gene	UCI MAKER Predicted Genes
3	BAM	IndCh_UCI_Illumina_Bowtie2	IndCh Illumina reads mapped on UCI using Bowtie2
		IndCh_UCI_Pacbio_CANU	IndCh Pacbio CANU corrected reads mapped on UCI
		UCIwg_IndchNI	IndCh whole genome mapped on UCI using minimap
4	VCF	IndCh_PB_UCI-BND	BND type structural variant predicted by PBSV
		IndCh_PB_UCI-CNV	Copy Number Variant structural variant predicted by PBSV
		IndCh_PB_UCI-DEL	Deletion structural variant predicted by PBSV
		IndCh_PB_UCI-INS	Insertion type structural variant predicted by PBSV
		IndCh_PB_UCI-INS_DUP	Insertion-Duplication type structural variant predicted by PBSV
		IndCh_PB_UCI-INV	Inversion structural variant predicted by PBSV
		IndCh_PB_UCI-SPLIT_DUP	Split-Duplication type structural variant predicted by PBSV
		UCI_PBSV	Structural variant predicted by PBSV with IndCh PacBio reads mapped on UCI reference
		SVMU_SV	Structural variant predicted by SVMU
5	BED	IndCh_UCI_BED	IndCh mapped on UCI

		UCI_Repeats_their	Repeats predicted for UCI from PMID: 33568145
6	Iso-seq BAM	R222-A01_aligned	Males (5-7 days old)
		R222-B01_aligned	Females (5-7 days old) unfed
		R222-C01_aligned	Females (3-6 hrs Post Blood Meal)
		R222-D01_aligned	Females (24 hrs Post Blood Meal)
		R223-A01_aligned	Females (48 hrs Post Blood Meal)
		R223-B01_aligned	Females (72 hrs Post Blood Meal)
7	BED	Physical_Marker	BLAST Physical Marker for the UCI genome

Supplementary Table 2 : Track details of JBrowse for UCI genome.

No.	File Type	Track Name	Track Content
1	FASTA	Reference sequence	IndCh genome as reference
2	GFF	Augustus_Gene	Genes predicted for IndCh genome using the 'Augustus' tool
3	BED	Physical_Marker	BLAST of physical markers on the IndCh assembly
		2RIIndchNI_REP	Repeat Elements predicted for IndCh
4	BAM	IndCh2R_UCI2	Chromosome 2 of UCI mapped on IndCh Chr 2R using minimap
		IndChNI_Illumina_ Bowtie	IndCh Illumina reads mapped on IndCh genome using 'bowtie2'
		IndCh_NI_PacBioC ANU	IndCh PacBio Canu corrected reads mapped on IndCh genome using 'minimap2'
		sorted_IndCh2R_Ast eiV4_Chr2R	Chr 2R of AsteI_V4 assembly mapped to IndCh Chr 2R using minimap

Supplementary Table 3 : Track details of JBrowse for the IndCh genome.

Sl. no.	Collection locations	Code	State	Life stage	Latitude, longitude coordinates	Month of collection
1.	Kannamangala, Bangalore	TI - Lab	Karnataka	Larva	13.21207°N, 77.72734°E	August 2016
2.	RR Nagar, Bangalore	B - Wild	Karnataka	Larva	12.9149°N, 77.5206°E	December 2018
3.	Annanagar, Chennai	TII - Lab	Tamil Nadu	Adult	13.018410°N, 80.223068°E	May 2016
4.	Dwarka, Delhi	TIII - Lab	Delhi	Larva	28.592140°N, 77.046051°E	December 2016
5.	Attavar, Mangalore	TIV - Lab	Karnataka	Larva	12.863348°N, 74.843258°E	December 2010
6.	Attavar, Mangalore	M - Wild	Karnataka	Larva	12.863348°N, 74.843258°E	December 2018

Supplementary Table 4 : GPS coordinates and collection date of An. stephensi population from different locations.

Sequence spanning IndCh chromosome 2R proximal breakpoint

CTTTGAAATGAATTAGATGATTTAAATCATCACGCAGCGTTATTTCAGCTCACTTTTGA
TTTAATTCGCCATGCCGATTAACCACTCACTCACTACTTTTTAACTCACTCACTATGAA
TGGCTCTCTC

Supplementary Text 1 : Highlighted in blue are the 78 bps in IndCh near the 2Rb proximal breakpoint.

Sequence spanning IndCh chromosome 2R distal breakpoint

ACACGGGTAAGAATATAATCCAATCATATCTTTAGCGAGCTGCAAATTAGTGATTTTC

Supplementary Text 2 : Highlighted in pink are the 8 bps in IndCh near the 2Rb distal breakpoint.
